# Tumor suppressor properties of the small C-terminal domain phosphatases in non-small cell lung cancer

**DOI:** 10.1042/BSR20193094

**Published:** 2019-12-13

**Authors:** George S. Krasnov, Grigory A. Puzanov, Marina A. Afanasyeva, Erdem B. Dashinimaev, Khava S. Vishnyakova, Artemy D. Beniaminov, Alexei A. Adzhubei, Tatiana T. Kondratieva, Yegor E. Yegorov, Vera N. Senchenko

**Affiliations:** 1 Laboratory of Postgenomic Research, Laboratory of Structural and Functional Genomics, Laboratory of Cellular Basics of Cancer Development, Laboratory of DNA-Protein Interactions, Laboratory of Protein Conformational Polymorphism in Health and Disease, Center for Precision Genome Editing and Genetic Technologies for Biomedicine. Engelhardt Institute of Molecular Biology, Russian Academy of Sciences, Moscow, Russia; 2 Laboratory of Cell Biology, Koltzov Institute of Developmental Biology, Russian Academy of Sciences, Moscow, Russia; 3 Research Institute of Clinical Oncology, Blokhin National Medical Research Center of Oncology, Russian Ministry of Health, Moscow, Russia; 4 Center for Genome Technologies, Pirogov Russian National Research Medical University, Moscow, Russia

**Keywords:** cell cycle, microRNA, SCP/CTDSP phosphatases, tumor suppressors

## Abstract

Non-Small Cell Lung Cancer (NSCLC) is responsible for the majority of deaths caused by cancer. Small C-terminal domain (CTD) phosphatases (SCP), CTDSP1, CTDSP2 and CTDSPL (CTDSPs) belong to SCP/CTDSP subfamily and are involved in many vital cellular processes and tumorigenesis. High similarity of their structures suggests similar functions. However their role in NSCLC remains insufficiently understood. For the first time we revealed the suppressor function of CTDSPs leading to a significant growth slowdown and senescence of A549 lung adenocarcinoma (ADC) cells *in vitro*. Their tumor-suppressive activity can be realized through increasing the proportion of the active form of Rb protein dephosphorylated at Ser^807/811^, Ser^780^, and Ser^795^ (*P*<0.05) thereby negatively regulating cancer cell proliferation. Moreover, we observed that a frequent (84%, 39/46) and highly concordant (Spearman’s rank correlation coefficient (*r_s_*) = 0.53–0.62, *P*≤0.01) down-regulation of *CTDSPs* and *RB1* is characteristic of primary NSCLC samples (*n*=46). A clear difference in their mRNA levels was found between lung ADCs with and without lymph node metastases, but not in squamous cell carcinomas (SCCs) (*P*≤0.05). Based on The Cancer Genome Atlas (TCGA) data and the results obtained using the CrossHub tool, we suggest that the well-known oncogenic cluster miR-96/182/183 could be a common expression regulator of *CTDSPs*. Indeed, according to our qPCR, the expression of *CTDSPs* negatively correlates with these miRs, but positively correlates with their intronic miR-26a/b. Our results reflect functional association of CTDSP1, CTDSP2, and CTDSPL, expand knowledge about their suppressor properties through Rb dephosphorylation and provide new insights into the regulation of NSCLC growth.

## Introduction

Lung cancer is the most commonly diagnosed cancer in the world, with high mortality [1]. Alterations in many cellular processes and signaling pathways contribute to tumorigenesis, and disruption of cell cycle regulation is one of the most crucial among them [2]. During the progression of cell cycle, retinoblastoma protein (Rb) undergoes (de)phosphorylation at multiple positions, which leads to changes in its activity and the ability to bind proteins such as E2F transcription factor [3,4]**.** The small C-terminal domain (CTD) phosphatases (SCP) – CTDSP1 (2q35), CTDSP2 (12q14.1), CTDSPL (3p22.2) and CTDSPL2 (15q15.3), belong to SCP/CTDSP subfamily of the haloacid dehalogenase (HAD) superfamily [5]**.** These proteins contain a catalytic F-cell production 1 (FCP1) homology domain that functions as a Ser/Thr phosphatase [6]. CTDSPs 1, 2, and L demonstrate 83–85% protein sequence homology of the central region (100–240 a.a.) and significant structural similarity of active centers (root-mean-square deviation, RMSD ∼0.6Å) [7]. Their catalytic centers also have high structural similarity, implicating similar functions [8]. CTDSPL2 has less sequence similarity to CTDSP1/2/L: 28% matches for full-sequence alignment and 65% for FCP1 homology domains only (see Supplementary Figure S1). The members of the SCP subfamily are involved in vital biological processes, which are often disrupted in cancer.

The *CTDSPL* (also known as *SCP3, HYA22, RBSP3, C3orf8*) has been first characterized as a tumor suppressor gene (TSG). It is often inactivated in various primary tumors and cell lines (renal, cervical, lung etc.) [9–12]. *In vitro* and *in vivo* experiments demonstrated its cell and tumor growth-inhibiting activities. The *CTDSPL* transient expression results in drastic reduction in Rb phosphorylated form (pRb) thus blocking the cell cycle at the G_1_/S boundary [9]. CTDSP1 (also known as NIF3, SCP1), negatively regulates cancer cell proliferation. It is a potential tumor suppressor for liver cancers, which acts through dephosphorylation of c-Myc at Ser^62^ [13]. CTDSP1 is able to block epithelial–mesenchymal transition (EMT) and to suppress cell migration by reversing MAPK-induced phosphorylation of the Twist-related protein 1 transcription factor [14]. *CTDSP*2 (also known as *OS4, PSR2*, and *SCP2*) is located in the genomic region that is frequently amplified in sarcomas and brain tumors [15]. *CTDSP1/2/L* are down-regulated in liver cancer [16]. In contrast, there are only few data about *CTDSPL2* (also known as SCP4) expression and functions in cancer cells. CTDSPL2 was identified as a common integration site in ALV-induced B-cell lymphomas, suggesting its potential role in driving oncogenesis [17].

Despite the great interest in SCPs in recent years, their role in lung cancer remains poorly understood. Our objective was to reveal functional associations between close members of the SCP subfamily in non-small cell lung cancer (NSCLC) using an integrated approach**.** Identification of their tumor suppressor activity would expand our knowledge about diverse pathways leading to lung cancer.

## Materials and methods

### Tissue specimens, clinical and pathological characteristics

A total of 46 NSCLC samples along with the adjacent morphologically normal tissue were obtained after surgical resection of tumors prior to radiation or chemotherapy and characterized according to the International TNM Classification system [18] in the Blokhin National Medical Research Center of Oncology of the Russian Ministry of Health, Russia. The clinical diagnoses were confirmed by pathomorphological examination at the Department of Tumor Pathologic Anatomy, Research Institute for Clinical Oncology, Moscow, Russia. Written informed consent was obtained from all patients. The use of clinical specimens for research purposes was conducted in accordance with the Declaration of Helsinki and approved by the Ethical Committee of Blokhin National Medical Research Center of Oncology. The clinicopathologic characteristics of the samples are summarized in Supplementary Table S1.

### Cell culture

A549 is an adenocarcinoma (ADC) cell line derived from human alveolar basal epithelial cells [19]. It was kindly provided by Dr. Maria Kost-Alimova (Karolinska Institute, Sweden). Cells were grown in DMEM medium (PanEco, Russia) supplemented with 10% fetal bovine serum (FBS; HyClone, U.S.A.), 2 mM l-glutamine (PanEco) and 40 μg/ml gentamycin (PanEco) in the atmosphere containing 5% CO_2_ at 37°C.

### Cell transfection and plasmids

To obtain stably transfected A549 cells, we used the Sleeping Beauty transposase (SB100) [20]. The pCMV(CAT)T7-SB100 vector was a gift from Dr. Zsuzsanna Izsvak (Addgene plasmid # 34879) and pT2/HB was a gift from Dr. Perry Hackett (Addgene plasmid # 26557). The coding sequences of *CTDSP1, 2*, or *L* were inserted into the pT2/HB plasmid (cloning was conducted by Evrogen, Moscow, Russia). The resulting plasmids (Supplementary Figure S2A), were transfected into A549 cells together with the pSB100 vector encoding the transposase, and pTagRFP vector (FP141, Evrogen, Russia) encoding red fluorescent protein (RFP) using Bio-Rad X-Cell Electroporation System. Next day after transfection, RFP-expressing cells were sorted using S3 cell sorter (Bio-Rad) and cloned by limiting dilution into 96-well plates (Costar, U.S.A.).

Alternatively, protein-coding DNA sequences of the genes *CTDSP1, 2*, or *L* were joined with the enhanced green fluorescent protein (EGFP) gene coding sequence through the T2A linker and cloned into the pT2/HB vector (Supplementary Figure S2B). It allowed us to measure the expression of the three proteins by EGFP fluorescence. Then, A549 cells were transfected with the resulting constructs together with pSB100 using Bio-Rad X-Cell Electroporation System.

### Measurement of growth rates of individual clones and ‘green’ cells

From 18th to 36th day, the clones of transfected cells were trypsinized and subcultured and the cells were counted. In parallel, the same procedure was carried out upon A549 cells transfected with pTagRFP only. Growth rate was calculated according to the following formula: V = log_2_ N/t, where V – growth rate (doubling a day), t – time (days), N – quantity of cells. In the EGFP co-expression approach, we performed cell sorting 24 h after transfection using the S3 cell sorter (Bio-Rad). After sorting, EGFP-expressing ‘green’ cells were counted and plated into wells of 24-well plates (10000 cells per well). After 5 days, the cells were counted again to assess growth rate.

### Colony formation assay

Сells were counted using a hemacytometer, and 200 cells were seeded into a 10-cm Petri dish (Costar, U.S.A.) and grown in DMEM medium (PanEco, Russia) supplemented with 15% FBS (HyClone, U.S.A.) in the presence of 5% СО_2_. On day 7, cells were fixed with 70% ethanol and stained with 0.1% Methylene Blue. This was followed by quantitative analysis of colonies using a stereomicroscope. The cell count of a colony was rounded up to the nearest bigger 2^n^ number. For example, if the number of cells in a colony was three or four, it was recorded as 4; if it was five to eight – as 8; if 129 to 256 – as 256.

Determination of senescence-associated β-galactosidase activity was performed as described in [21]. Cells were washed three times with PBS (pH 7.2), fixed with 0.4% glutaraldehyde in PBS (pH 7.2), washed three times for 5 min each with PBS (pH 7.2), and stained in fresh, filtered X-gal solution (1 mg/ml X-gal, 3 mM K_3_Fe(CN)_6_, 5 mM K_4_Fe(CN)_6_, 1 mM MgCl_2_, 1% Nonidet-P40 in PBS, pH 6.0) overnight at 37°C.

### Western blot assay

Cells were lysed in radio-immunoprecipitation assay (RIPA) buffer (150 mM sodium chloride, 1% NP-40, 0.5% sodium deoxycholate, 0.1% SDS, 50 mM Tris, pH 8.0) supplemented with phosphatase and protease inhibitors (1 mM PMSF, 1 mM sodium orthovanadate). Total protein concentration was determined by Bradford’s assay. After reducing SDS/PAGE, proteins were blotted on to nitrocellulose membrane by wet transfer. Total pRb and its phosphorylated forms were measured using Rb Antibody Sampler Kit (Cell Signaling Technology, cat. no. 9969) according to the manufacturer’s protocol. SignalFire™ ECL Reagent (Cell Signaling Technology, cat. no. 6883) was used for detection.

### Enzyme-linked immunosorbent assay

Cell lysates were diluted to 10 ng/ml with PBS, loaded into the wells of microtiter plates (100 μl per well), and incubated overnight at 4°C. Wells were blocked with 5% BSA in PBS for 2 h at room temperature and incubated with monoclonal antibodies against total and phosphorylated Rb (Cell Signaling Technology, cat. no. 9969) diluted 1:1000 in PBS for 1 h at 37°C. After washing, HRP–conjugated secondary antibodies (Cell Signaling Technology, cat. no. 7074 and 7076) diluted 1:1000 in PBS were added and incubated for another 1 h at 37°C. After incubation with 100 μl of TMB chromogenic substrate (Elabscience, E-IR-R201) for 30 min, the reaction was stopped with 100 μl H_2_SO_4_ (0.18 M). Absorbance at 450 nm was measured using Multiskan EX (Thermo Fisher Scientific). Plates were washed four times with washing buffer (PBS pH 7.4 containing 0.1% (v/v) Tween 20) after each step. The values represent mean of eight wells.

### Bioinformatics analysis

We used original CrossHub tool (https://sourceforge.net/projects/crosshub/) to evaluate expression alterations of the members of SCP subfamily and *RB1* in NSCLC to reveal common mechanisms of their expression regulation [22]. CrossHub enables the analysis of the multidimensional The Cancer Genome Atlas (TCGA) data on 20+ human tumor types including gene/miRNA expression. We focused on two datasets, lung ADC and squamous cell carcinoma (SCC). Each dataset included approximately 350–500 tumor and 40–60 adjacent normal tissue samples for RNA-Seq and miRNA-Seq analyses. Briefly, to identify potential miRNA regulators of these genes with CrossHub, we combined the results of RNA-Seq – miRNA-Seq co-expression analysis and the predictions of miRNA binding site. The expected regulatory microRNAs should fit the following criteria: (1) have miRNA binding site(s) that were predicted with several algorithms (TargetScan, DIANA microT etc.); (2) demonstrate mRNA–microRNA expression anti-correlation; (3) be up-regulated in tumors and have sufficient expression levels.

### RNA extraction and qPCR analysis

Liquid nitrogen-frozen tissues were disrupted using a MagNA Lyser Instrument (Roche) and MagNA Lyser Green Beads (Roche). Total RNA was extracted using miRneasy Mini Kit (Qiagen, cat. no. 217004) and reverse transcribed using random hexamer primers and RevertAid reverse transcriptase (Thermo Fisher Scientific, cat. no. EP0441). The mRNA levels of target genes and miRNAs were measured by qPCR using TaqMan Gene Expression Assays (Applied Biosystems CA, U.S.A.): CTDSP1, CTDSP2 (Assay ID: Hs01105503_m1, Hs00938977_m1); hsa-miR-26a, hsa-miR-26b, hsa-miR-183, hsa-miR-96, hsa-miR-182 (Assay ID: 000405, 000407, 002269, 000186, 002334); RNU6, RNU48 (Assay ID: 001093, 001006). The set of primers and TaqMan probes are listed in Supplementary Table S2. Relative mRNA levels were calculated according to the ΔΔ*C*_t_ method [23] using the reference genes *RPN1, RNU6* and *RNU48.*

### Statistical analysis

Nonparametric Wilcoxon test was used to compare mRNA level differences of target and reference genes in the NSCLC samples. Kruskal–Wallis and Mann–Whitney rank-sum tests were used for analysis of the mRNA level changes in tumor groups with different histological characteristics. *P-*values <0.05 were considered statistically significant. Spearman’s rank correlation coefficient (*r*_s_) was used for revealing correlations between relative mRNA levels of the studied genes and miRNAs.

## Results

### Differential expression of SCP subfamily genes and RB1 in NSCLC (TCGA data)

**Figure 1:**
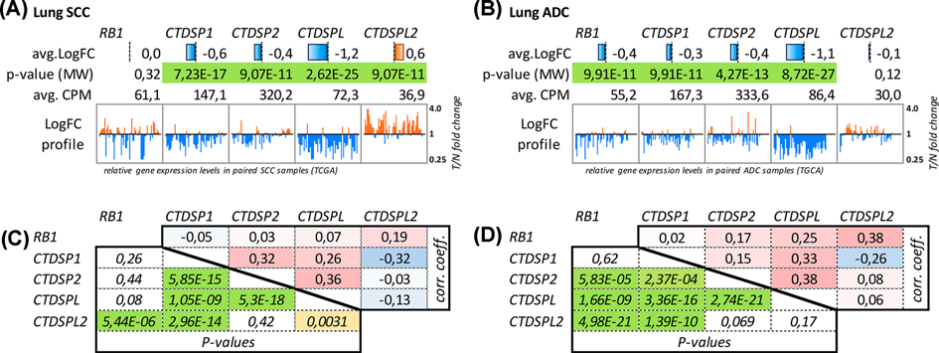
Analysis of *RB1* and SCP subfamily genes expression in lung cancer (TCGA data) (**A**) SCC and (**B**) ADC. *Avg. LogFC* – binary logarithm of average expression level fold change (T/N, tumor versus adjacent normal tissue); avg. *CPM* – average read counts per million (pooled tumor and normal samples), which reflects the absolute gene expression level; *P-value (MW)* – statistical significance of T/N expression differences according to Mann–Whitney U-test; *LogFC profile* – T/N expression ratio (axis is geometrically log-transformed) across individual paired samples; (**C,D**) Co-expression of *RB1* and SCP subfamily genes in lung cancer (TCGA data). Upper diagonals of the matrices show Spearman’s rank correlation coefficients; lower diagonals – Spearman’s *P*-values. Cell colors are linked to cell values.

Using the CrossHub tool we analyzed the expression alterations of four members of the SCP subfamily *— CTDSP1/2/L/L2* and *RB1* genes in **NSCLC** (ADC, SCC). The most significant down-regulation was observed for *CTDSPL*, both in ADC and SCC, with two-fold or greater decrease in 70 and 50% samples, respectively (Figure 1, upper panels (A,B)). *CTDSP1* and *CTDSP2* demonstrated only slight under-expression tendency in both histological types (approximately 1.5-fold decrease on average; 2-fold or greater decrease was observed only in 7–20% samples). *CTDSPL2* did not show any tendencies in expression alterations in lung ADC but was characterized with overexpression in SCC.

Some of these genes demonstrated statistically significant co-expression patterns: *CTDSPL—CTDSP1/2* and *CTDSPL—RB1* in ADC, and *CTDSPL—CTDSP1/2* in SCC (Figure 1, lower panels (C,D)) suggesting the possibility of common expression regulation mechanisms.

According to TCGA data, *CTDSP1, 2* and *L2* did not show any significant correlations with the N index (TNM-classification). There is only a slight association between *CTDSPL* expression and the presence of lymph node metastases (*r_s_* = – 0.1, *P*=0.02) in the case of ADC. Similarly, no such correlation was found in the SCC dataset.

Unlike *CTDSP1/2/L, CTDSPL2* expression was not decreased in tumors. Conversely, we observed frequent up-regulation of *CTDSPL2* expression, especially in lung SCC. *CTDSPL2* did not demonstrate positive co-expression (−0.32 < *r_s_* < 0.08) with any other member of the SCP subfamily, but did show positive correlation with *RB1* (*r_s_* = 0.38). This discordance of *CTDSPL2* and *CTDSL1/2/L* was expected, given the weaker homology of *CTDSPL2* to other members of the SCP subfamily (Supplementary Table S3). Therefore, we have excluded *CTDSPL2* from further experimental analysis and focused on *CTDSP1/2/L* and *RB1* genes.

**Figure 2:**
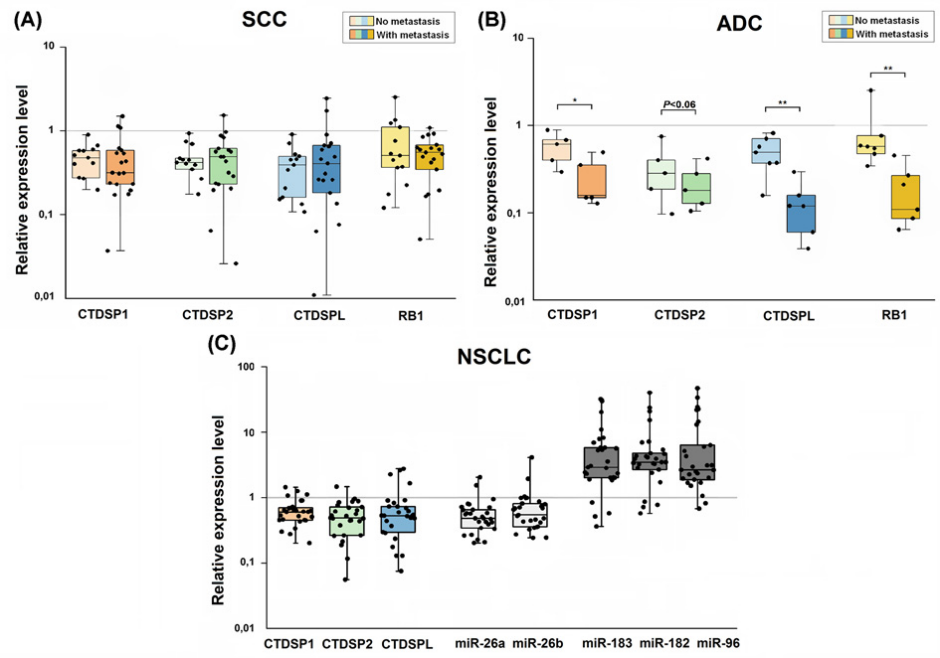
Quantitative real-time PCR analysis of *CTDSP1/2/L, RB1* and microRNA expression in NSCLC (**A,B**) Relative *CTDSP1/2/L* and *RB1* expression in metastatic and non-metastatic SCC (*n*=33) and ADC (*n*=13) normalized to *RPN* mRNA level. (**C**) *CTDSP1/2/L*, intronic miR26a/b, and miR-96/182/183 expression in NSCLC samples (*n*=28) normalized to *RPN1, RNU6*, and *RNU48* expression, respectively. The boxes represent the interquartile ranges (25–75th percentiles); horizontal lines inside the boxes indicate the medians. The vertical whiskers extend to the maximum and minimum values. *P≤0.05; **P≤0.01 (Mann–Whitney U-test).

Additionally, we evaluated expression levels of mir-26a/b (located in the introns of *CTDSP1/2/L* genes) according to TCGA data (miRNA-Seq) using the CrossHub tool. The results are presented in the Supplementary Table S4. Both of these microRNAs are slightly down-regulated (1.5–2.5-fold on the average) in both lung ADC and SCC, and this agrees with qPCR data (see Figure 2C).

### Simultaneous down-regulation of SCP subfamily genes and RB1 in NSCLC (qPCR data on an independent cohort)

qPCR analysis revealed noticeable (three- to seven-fold on average) and frequent (60–90%) mRNA level decrease for *CTDSP1/2/L* and *RB1* in NSCLC (SCC and ADC) samples. In addition, we showed a clear difference of the *CTDSP1, CTDSPL* and *RB1* relative mRNA levels in ADC with and without lymph node metastases, but not in SCC (*P*≤0.05) (Figure 2A,B; Supplementary Table S5). Moreover, expression levels of *CTDSP1/2/L* and *RB1* appeared to be highly correlated in SCC (*r_s_* = 0.50–0.73, *P*≤0.05), although the correlation for *CTDSP1* and *RB1* was not very strong (*r_s_*= 0.31, *P*=0.07). While in ADC, statistically significant co-expression was only observed for *CTDSPL, CTDSP1*, and *RB1* genes (Table 1A, Supplementary Figure S3A–G). In general, SCC was characterized by more pronounced co-expression of the genes than ADC.

**Table 1:** Spearman’s correlation coefficients (*r_s_*): qPCR (**A,C**) and TCGA RNA-Seq data (**B**) in NSCLC (ADC, SCC)

A	RB1	CTDSP1	CTDSP2
**Lung ADC (qPCR)**			
***CTDSPL***	0.67 (*P*=0.013)	0.67 (*P*=0.035)	0.55 (*P*=0.4)
***RB1***	–	0.54 (*P*=0.1)	0.20 (*P*=0.3)
***CTDSP1***	–	–	0.42 (*P*=0.2)
**Squamous cell lung cancer (qPCR)**			
***CTDSPL***	0.74 (*P*<0.001)	0.50 (*P*=0.003)	0.73 (*P*<0.001)
***RB1***		0.31 (*P*=0.07)	0.65 (*P*<0.001)
***CTDSP1***	–	–	0.73 (*P*<0.001)

B	*CTDSPL*		*CTDSP1*		*CTDSP2*	
	r_s_	algorithms	r_s_	algorithms	r_s_	algorithms
**Lung ADC (TCGA data)**						
***miR-96***	−0.26	D, Tn	−0.22	D, Tc	−0.29	D, Tc
***miR-182***	−0.22	D, Tn	−0.21	D, Tc, Tn	−0.18	Tc
***miR-183***	−0.27	D, Tc, Tn, Rw	−0.27	D, Tc, Tn	−0.17	Tn
**Squamous cell lung cancer (TCGA data)**						
***miR-96***	−0.34	D, Tn	−0.10	D,Tc	−0.28	D, Tc
***miR-182***	−0.16	D, Tn	−0.10	D,Tc,Tn,	−0.14	Tc
***miR-183***	−0.27	D, Tc, Tn, Rw	−0.24	D,Tc,Tn	−0.14	Tn
(*P*<0.01 for all cases)						

C	*CTDSPL*	*CTDSP1*	*CTDSP2*
**Non-small squamous cell lung cancer (qPCR)**			
***miR-96***	−0.26	−0.34 (*P*=0, 08)	−0.62 (*P*<0.001)
***miR-182***	−0.03	−0.24	−0.42 (*P*<0.05)
***miR-183***	-0.08	−0.16	−0.35 (*P*=0.065)
***miR-26a***	0.23	0.16	0.37 (*P*<0.05)
***miR-26b***	0.25	0.15	0.39 (*P*<0.05)
(*P*>0.05 for all cases, unless otherwise specified)			

(**A**) The comparison of the mRNA levels of *CTDSPs* and *RB1* in primary ADC and SCC samples. (**B**) Results of joint analysis of TCGA and microRNA target prediction resources performed by the CrossHub tool (https://sourceforge.net/projects/crosshub/). Algorithms: D – DIANA microT, Tc – TargetScan conservative sites, Tn – TargetScan non-conservative sites, Rw – miRTarBase weak evidence. (**C**) The comparison of the mRNA levels of *CTDSPs* and microRNAs in NSCLC.

Down-regulation of TSGs is one of the hallmarks of carcinogenesis [24]. The simultaneous and very frequent expression decrease in *CTDSP1/2/L* allowed us to assume that they harbor growth suppressing activity and suggest the existence of a common mechanism of their inactivation.

### *In vitro* cell growth suppression by CTDSP1, CTDSP2, and CTDSPL

**Figure 3:**
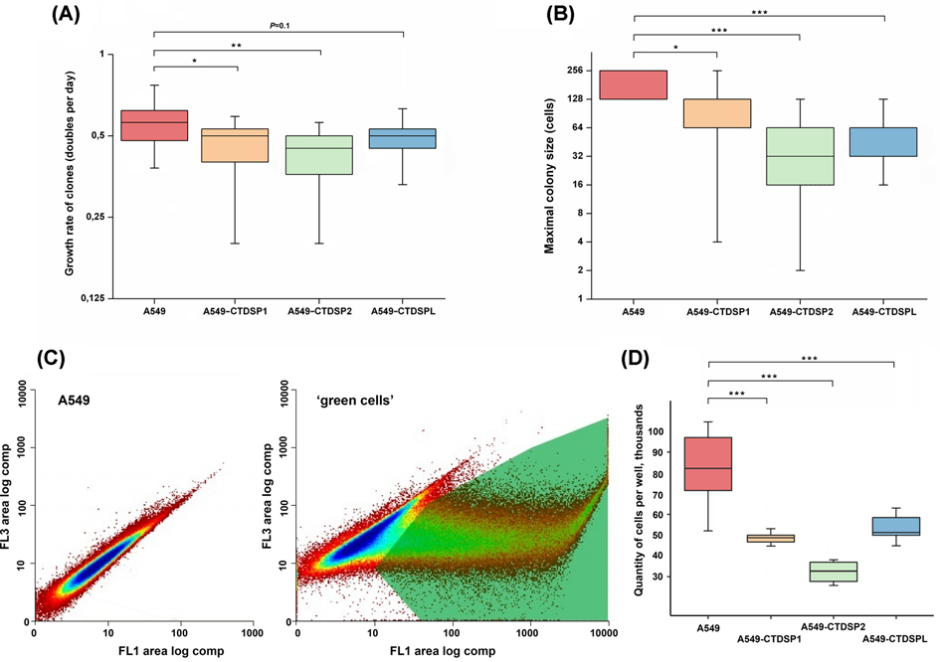
Suppression of the growth of A549 cells after transfection with CTDSP1/2/L (**A**) Growth rate of transfected clones. The boxes represent the interquartile range (25–75th percentiles); horizontal line inside the box indicates the median. The vertical whiskers extend to the maximum and minimum values. The gray line indicates the median value for non-transfected cells. (**B**) Distribution of the maximal sizes of colonies (colony formation assays). For A549 vertical whiskers are within the box. For A549, A549-CTDSP1 and A549-CTDSPL median coincides with the 25th percentile. (**C**) Fluorescence distribution of original A549 cells and transfected cells. FL3 (red) and FL1 (green). The gate for sorting is shown. (**D**) Suppression of ‘green’ cells growth, the box-and-whisker plot; the significance of differences of A549, transfected with the pSB100 plasmid only, is determined by the nonparametric Wilcoxon test. For (A,B,D), *P≤0.05; **P≤0.01; ***P≤0.001.

Protein-coding DNA sequences of the genes *CTDSP1/2/L* were cloned into the pT2/HB vector and transfected into A549 cells (Supplementary Figure S2). Before testing the ability of *CTDSP1/2/L* to suppress tumor growth *in vitro*, we evaluated the efficiency of incorporation of the transgenes in transfected clones – copy number (DNA) and expression level (mRNA**).** The efficiency of incorporation of the transgenes was 30.0%, (9/30), 35.7% (10/28), and 27.3% (12/44), respectively. Then we selectively evaluated the mRNA level of three transgenes in transfected cells relative to their mRNA levels in the parental A549 cells (control cells) and confirmed the overexpression (mean 1.8) of all three transgenes in the transfected cells compared with control. Despite the high heterogeneity of clones of both transfected and parental A549 cells, the growth rate calculation revealed that all three transgenes caused a decrease in the growth rate (Figure 3А)**.** In two cases, *CTDSP1* and *CTDSP2*, the suppression of growth was significant (*P*=0.04 and 0.007). Three of the slowest clones eventually (2 months later) completely ceased proliferation and were lost. Additionally, we evaluated the dynamics of colony formation by transfected clones. The maximum size of colonies was estimated. It turned out that all three constructs significantly decreased the maximal colony size (Figure 3B).

EGFP co-expression confirmed these data. In this case expression of transgenes was accompanied by equimolar expression of the fluorescent green marker. This allowed us to separate cells with high level of expression of the three transgenes early after transfection by flow sorting, and to reveal suppression of cell growth and the status of phosphorylation of Rb. The experiments with ‘green’ cells were finished in 6 days only. Twenty-four hours after transfection, we sorted transgene-expressing cells by their green fluorescence (Figure 3C), and determined their growth rate during the next 5 days. The efficiency of transfection determined by green fluorescence was 41.1, 38.7, 40.0% for CTDSP1, CTDSP2, and CTDSPL, respectively. The significance of the differences (*P*<0.001 (Figure 3D)) prove that transgenes actually inhibited cell growth.

### Morphological changes of A549 cells after transformation

**Figure 4:**
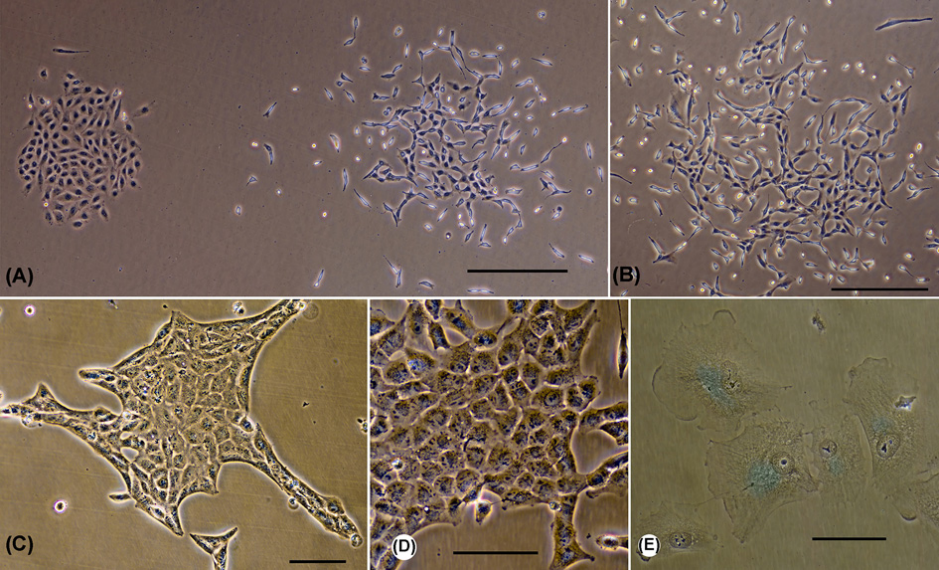
Morphological types of colonies of A549 cells and their transfectants (**A**) Two neighboring colonies of A549 cells have very different morphologies. The left represents an islet of contacting cells with a morphology intermediate between epithelial cells and fibroblasts. The cells of the right colony are strongly elongated. These cells have classical fibroblast morphology and have increased motility. (**B**) Colony of A549 cells with prominent ‘fibroblastic-migratory’ morphology. (**C**) Colony of A549 transfectants, having a shape of an islet of epithelioid cells. The edges of the islet are formed by special ‘marginal’ cells. (**D**) Colony of A549 transfected cells, consisting of an islet of epithelioid cells. (**E**) Senescent cells in the colony. Blue color – senescence-associated β-galactosidase staining. (A,B) The parental A549 line. (C–E) Transfected cells (C,D: CTDSP2, E: CTDSPL). Scale bars: (A,B) – 500 µm, (C–E) – 100 µm. Phase-contrast, digital contrast.

The A549 cells are highly heterogeneous [25]. According to our data, they can form colonies of completely different morphologies in close proximity to one another. Some colonies consist mainly of epithelioid cells. These cells are flat, well-spread, not elongated, and stay in direct contact with each other (left colony at Figure 4A). These cells also have low mobility. Other colonies consist of elongated cells, which are not spread and interact less with the substrate (right colony at Figure 4A). These cells are more mobile, and the area of intercellular contacts is reduced. All these features are characteristic of the fibroblastoid phenotype. An extreme variant of such a ‘fibroblastic-migratory’ phenotype can be seen in Figure 4B. Such colonies were observed only for the parental A549 cells. Among the transfectants, reinforced epithelioid phenotypes were often found, as shown in Figure 4C,D. Figure 4D demonstrating an ‘open’ colony, in which all cells are of similar morphology. Figure 4C shows a different colony type, with the outer cells forming a clear boundary, which indicates an obstacle in the growth of the colony and an increase in intercellular interaction. Some colonies of the transfected cells underwent senescence (Figure 4E). However, this is not their exclusive feature and could also be observed as a very rare event in the parental line. Thus, morphological analysis of cell showed that all three constructs, in addition to suppressing growth, cause an increase in the proportion of senescent cells and cells with epithelial morphology.

### Transient expression of CTDSP1/2/L leads to decreased phosphorylation of Rb at Ser^807/811^, Ser^780^, and Ser^795^

**Figure 5:**
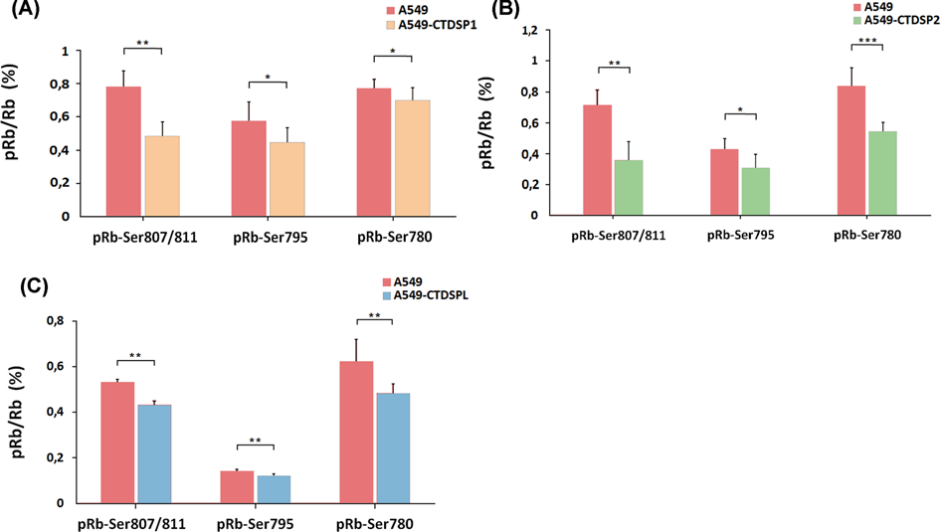
Ratio of phosphorylated Rb (pRb) after transfecting A549 cells with plasmids encoding either CTDSP1 (A), CTDSP2 (B), or CTDSPL (C) In all three cases, the ratio of phosphorylated Rb protein is decreased after transfection. pRb/Rb, relative amount of pRb normalized to the total Rb protein. Intact A549 were compared with the slowest growing clones of A549 stably transfected with pT2/HB-СTDSP1, 2 or L. A minimum of two independent ELISA experiments were performed. The data represent the means of 8 wells ± SD. *P≤0.05; **P≤0.01; ***P≤0.001 (Mann–Whitney U-test).

Western blot data showed a decrease in Rb phosphorylation at Ser^807/811^ in A549 cells stably transfected with plasmids encoding either *CTDSP1, CTDSP2*, or *CTDSPL* (Supplementary Figure S4). To confirm this observation, we analyzed A549 clones obtained by stable transfection with plasmids containing the same genes fused with EGFP through T2A linker, using more sensitive enzyme-linked immunosorbent assay (ELISA), to evaluate Rb phosphorylation status of the three sites Ser^807/811^, Ser^795^, and Ser^780^ (Figure 5). All these sites are located in the C-terminal tail of the pocket domain and are modified by Cdk during cell cycle progression [26]**.**

Using ELISA, a decrease in Rb phosphorylation level after transfection with pT2/HB-CTDSP1, 2, or L was detected for all three phosphorylation sites. The most significant changes were observed for Ser^780^ and Ser^807/811^ (Figure 5). The reduction in phosphorylation level was the strongest in cells transfected with pT2/HB-CTDSP2. It reached almost 50 and 35% for Ser^807/811^ and Ser^780^, respectively, while for A549-CTDSP1 and A549-CTDSPL it comprised approximately 38 and 19% at Ser^780^, and only 10 and 22% at Ser^780^, respectively. Thus, our results confirm the ability of CTDSPL/1/2 to change the phosphorylation status of Rb in A549 cells thereby affecting the progression of the cell cycle.

### Bioinformatics prediction of microRNAs that could target SCP phosphatase genes and RB1 in NSCLC (TCGA data)

To identify potential common microRNA that target *CTDSP1/2/L* and *RB1* genes, we used CrossHub tool and TCGA data. The presence of miRNA binding site (predicted with several algorithms) at 3′-UTR of a specific mRNA and the negative mRNA–miRNA expression correlation suggests that the mRNA can be coregulated with such miRNA**.** Surprisingly, the analysis with CrossHub revealed that all the members of a well-known oncogenic cluster—miR-96, miR-182, and miR-183, take first places in common microRNA rating for lung SCC and ADC (Supplementary Tables S6 and S7). These microRNAs have negative miRNA–mRNA expression correlation coefficient with *CTDSP1/2/L* (*r*_s_ values range from −0.17 to −0.27, *P*-values range from 10^−3^ to 10^−7^) in SCC and with *RB1* and *CTDSP1/2/L* in ADC (*r*_s_ values range from −0.1 to −0.34, *P-*values range from 0.03 to 10^−23^). The presence of a binding site for at least one of these microRNAs has been predicted using several algorithms for *CTDSP1/2/L* but not for *RB1* and *CTDSPL2* genes. All these microRNAs, miR-183, miR-182, and miR-96, are overexpressed in tumors compared with normal tissue (four- to seven-fold) both in ADC and SCC, according to the TCGA data. Among them, miR-96 has the lowest expression level compared with miR-182 and miR-183 significantly lower expression level (Supplementary Table S6 and S7). This implies that miR-182 and miR-183, but not miR-96, are more likely to represent regulators of *CTDSP1/2/L* genes in NSCLC.

### Quantitative comparative analyses of CTDSP1/2/L, miR-96/182/183 and miR-26a/b expression in NSCLC (qPCR data on an independent cohort)

Negative correlations between the expression of oncogenic cluster miR-96/182/183 (overexpression) and target genes *CTDSP1, CTDSP2* and *CTDSPL* (down-regulation) were found (Figure 2C, Table 1B,C). The strongest negative correlation was noticed for miR-183 and *CTDSP2* (*r_s_*= −0.62, *P*<0.001). The average value of the miR-96 expression level in both normal and tumor samples (relative to the reference small-nucleolar RNA *RNU48*) is much lower than that of miR-182 and miR-183. This is in agreement with TCGA miRNA-Seq data described above. Nevertheless, the relative (tumor-to-normal) expression levels of all three microRNAs were significantly increased (five- to six-fold on average) in the majority of samples (79–82%) according the qPCR data (Figure 2C). Moreover, there was a very high correlation (*r*_s_ = 0.73–0.79, *P*≤0.001) between the relative expression levels of all three miRNAs in NSCLC samples (Supplementary Figure S3H–J). Thus, our results suggest the involvement of miR-96/182/183 cluster in the regulation of Rb protein activity via inactivating *CTDSP1/2/L* expression in NSCLC.

At last, we evaluated the expression of intronic miR-26a and miR-26b along with their host genes *CTDSP1/2/L* (Supplementary Figure S5)*.* Co*-*expression (down-regulation) of *CTDSP1/2/L* and miR-26a/b were found in the majority of NSCLC samples (Figure 2C). For comparison, *CTDSP1/2/L* mRNA level decreased three- to seven-fold on average (60–90% samples) and miR-26a/b level decreased 2.7-fold on average (61–75% samples). The strongest correlations (0.37 and 0.39, *P*<0.05) were found between miR-26a, miR-26b, and *CTDSP2* (Table 1C). This result indicates that *CTDSP1/2/L* and miR-26a/b are unlikely antagonists in NSCLC. However, the magnitude of expression level decrease in these miRNAs and mRNA was different, which may result from post-transcriptional events.

## Discussion

Despite significant achievements in the diagnostics and treatment of lung cancer during recent years, it still remains important to search for new participants of the protective mechanisms that prevent malignant transformation of human lung cells. New approaches to antitumor therapy include simultaneous use of multiple targets to block or, conversely, restore their functions [27]**.** At present, multifunctional proteins of the SCP subfamily and their genes can exhibit tumor suppressor or oncogenic activity, depending on the tumor type. *CTDSPL* gene was the major object of our studies for a number of years. Earlier, its tumor suppressor properties were described both *in vitro* and *in vivo* using KRC/Y (renal cancer) and MCF-7 (breast cancer) cell lines [9]. Later, we demonstrated direct interaction of CTDSPL and Rb using surface plasmon resonance spectroscopy [28]**.** A number of other studies addressed tumor suppressor activity and clinical prognostic significance of genomic and transcriptomic alterations of *CTDSPL* in different types of cancer [10–12,29]**.** Another member of the SCP subfamily, CTDSP1, suppressed proliferation, migration, and invasion of kidney cancer cells *in vivo* [30]. Overexpression of *CTDSP2* reduced the number of cells in S-phase and inhibited cell cycle progression in a bone osteosarcoma cell line [31]. Altogether, these data suggest tumor suppressor activity of *CTDSPL, CTDSP1*, and *CTDSP2* genes in different types of cancer. However, the close homologs are still being studied separately, primarily, in lung cancer.

Our expression data (qPCR) are consistent with the results of TCGA RNA-Seq analysis: we observed frequent and highly coordinated down-regulation of *CTDSP1/2/L* and *RB1* in primary lung tumors. In addition, we noticed a clear difference in their mRNA levels between ADC with and without lymph node metastases (but not for SCC; Figure 2 and Table 1), which suggests that these genes could be used as biomarkers of the ADC metastatic status.

For the first time we confirmed tumor suppressor properties of all three phosphatases, CTDSP1/2/L, in the A549 lung ADC cell line *in vitro* (Figures 3 and 4). In addition, tumor suppressive activity of CTDSP1/2/L appears to be mediated through dephosphorylation of at least three serine residues of Rb (Ser^807/811^, Ser^780^, and Ser^795^ for each protein) *in vitro*. Rb protein belongs to the ‘pocket proteins’ family, containing several protein binding domains, and is capable of interacting with E2F transcription factors and c-Abl tyrosine kinase [32]. Each of the binding domains is inhibited by phosphorylation at different sites. These sites are phosphorylated by CDK2-CyclinE and CDK4-CyclinD before entering S-phase [32–34]. Ser^795^ is one of those sites that undergoes hyperphosphorylation by CDK2-E in late G_1_ phase, which leads to the inhibition of Rb binding to E2F and finally to increased expression of S-phase genes [35]. Contrary to Ser^795^, Ser^780^, and Ser^807/811^ are exclusively phosphorylated by CDK4-D [32,34]. Phosphorylation at Ser^807/811^ is necessary for the inhibition of Rb interaction with c-Abl [32]. Dephosphorylation of Rb at Ser^780^ and Ser^795^ sites can lead to its activation and ability to bind E2F transcription factors, which may conduce to the observed effect of suppressing the growth of A549 tumor cells upon transfection with vectors containing *CTDSP*1/2/L genes. Dephosphorylation of the Ser^807/811^ site is a necessary modification for the ability of Rb to bind the c-Abl tyrosine kinase that phosphorylates Ser^5^ of the CTD of RNA Pol II largest subunit and thereby enhances its activity [32]. Thus, by increasing the expression of the *CTDSP1/2/L* genes during the G_1_ phase and decreasing it in the S-phase, the activity of RNA Pol II is inhibited during G_1_ and restored during S-phase [16].

In summary, we demonstrate that CTDSP1/2/L genes are important regulators of the cell cycle, which possed anti-proliferative activity. The simultaneous down-regulation of these genes in NSCLC suggests the presence of common mechanisms of their regulation. Previously, using NotI-microarrays we found that the frequency of *CTDSPL* mRNA level decrease in both SCC and ADC was much higher than the frequency of CTDSPL deletions and/or methylation [36]. This suggests the presence of other mechanisms besides promoter hypermethylation.

To uncover these common regulatory mechanisms, we applied the CrossHub tool, which enables multi-way analysis of TCGA data (gene and miRNA expression, methylation profiling, miRNA and transcription factor targets prediction). We did find promoter hypermethylation (especially of *CTDSPL* gene) but its frequency was insufficient to explain the observed down-regulation of expression (data not shown). Hence, we focused on microRNA interference and found that an oncogenic microRNA cluster—miR-96/182/183, can act as possible expression suppressor for CTDSP1/2/L. Expression of this microRNA family is deregulated in different types of cancer. MiR-96/182/183 cluster plays dual role, either oncogenic, pro-metastatic, or tumor suppressive, depending on the type of tumor [37–40]. Co-expression of miR-183-96-182 was demonstrated in some tissues and diseases, including cancer [37]**.** Interestingly, the greatest negative correlation with miR-96/182/183 was seen for *CTDSP2*, which also had the greatest suppressive effect on A549 proliferation. Other potential common microRNA regulators of *CTDSP1/2/L* expression are miR-9 and miR-149 for SCC, and miR-9, miR-503, and miR-193b for ADC (Supplementary Tables S6 and S7).

*CTDSP1/2/L* are host genes for miR-26a1/2 (intronic miRNA of *CTDSPL* and *CTDSP2*) and miR-26b (intronic miRNA of *CTDSP1*) (Supplementary Figure S5). The miR-26a/b and their host genes can exhibit synergistic and antagonistic properties relative to each other. This exerts different effects on cell migration and invasion capabilities [41–43]**.** *CTDSP1/2/L* and miR-26a/b are co-expressed in concert with cell cycle phases in hepatocellular carcinoma [16]. However, miR-26a inhibits the expression of *CTDSP2* in neuroblastoma cell line *in vitro* [44]**.** According to TCGA and our quantitative data, *CTDSPL/1/2* and miR-26(a/b) are co-expressed (down-regulated) in NSCLC. Taking into account the available literature, such a unidirectional change in expression may indicate their cooperation and probably common functions in cell cycle suppression of lung cancer cells.

**Figure 6:**
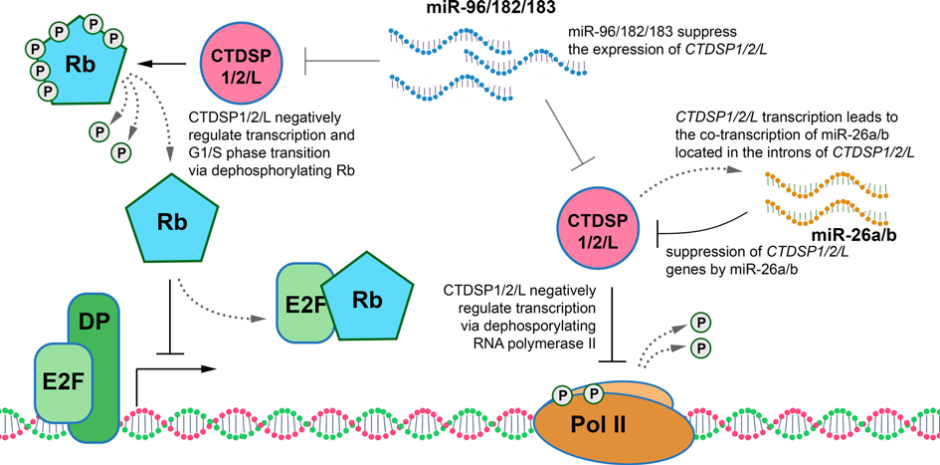
Schematic representation of the possible roles of CTDSP1/2/L, miR26a/b, and miR-96/182/183 genes in the regulation of the cell cycle in NSCLC DP – transcription factor that forms a heterodimer with E2F.

In sum, our data clarify the role of SCP phosphatases in the pathogenesis of NSCLC, suggest possible mechanism of their deregulation and expand our knowledge of how CTDSP1/2/L can participate in the regulation of the cell cycle (Figure 6). Identification of tumor suppressors with similar properties and functions, such as SCP phosphatases that co-operate in protecting lung cells from malignant transformation, can aid the development of new therapeutic strategies for NSCLC.

## Supplementary Material

Supplementary Figures S1-S5 and Tables S1-S7

## Abbreviations

ADC: adenocarcinoma
CTD: C-terminal domain
CTDSP: CTD small phosphatase
CTDSPL: CTD phosphatase-like
*CTDSP1/2/L* or *CTDSP*: *CTDSP1, CTDSP2* and *CTDSPL* genes
EGFP: enhanced green fluorescent protein
ELISA: enzyme-linked immunosorbent assay
FBS: fetal bovine serum
FCP1: F-cell production 1
NSCLC: non-small cell lung cancer
Rb, *RB1*: retinoblastoma protein, and its gene
pRb: phosphorylated forms of Rb
SB100: Sleeping Beauty transposase
SCC: squamous cell carcinoma
SCP: small C-terminal domain phosphatase
TCGA: The Cancer Genome Atlas
TSG: tumor suppressor gene
*r_s_*: Spearman’s rank correlation coefficient

## References

[1] Ferlay J., Soerjomataram I., Dikshit R., Eser S., Mathers C., Rebelo M. et al. (2015) Cancer incidence and mortality worldwide: sources, methods and major patterns in GLOBOCAN 2012. Int. J. Cancer 136, E359–E386 10.1002/ijc.29210 25220842

[2] Eymin B. and Gazzeri S. (2010) Role of cell cycle regulators in lung carcinogenesis. Cell Adh. Migr. 4, 114–123 10.4161/cam.4.1.10977 20139697 PMC2852568

[3] Wang J.Y., Knudsen E.S. and Welch P.J. (1994) The retinoblastoma tumor suppressor protein. Adv. Cancer Res. 64, 25–85 10.1016/S0065-230X(08)60834-9 7879661

[4] Burkhart D.L. and Sage J. (2008) Cellular mechanisms of tumour suppression by the retinoblastoma gene. Nat. Rev. Cancer 8, 671–682 10.1038/nrc2399 18650841 PMC6996492

[5] Zhang M., Liu J., Kim Y., Dixon J.E., Pfaff S.L., Gill G.N. et al. (2010) Structural and functional analysis of the phosphoryl transfer reaction mediated by the human small C-terminal domain phosphatase, Scp1. Prot. Sci. 19, 974–986 20222012 10.1002/pro.375 PMC2868240

[6] Yeo M., Lin P.S., Dahmus M.E. and Gill G.N. (2003) A novel RNA polymerase II C-terminal domain phosphatase that preferentially dephosphorylates serine 5. J. Biol. Chem. 278, 26078–26085 10.1074/jbc.M301791200 12721286

[7] Kamenski T., Heilmeier S., Meinhart A. and Cramer P. (2004) Structure and mechanism of RNA polymerase II CTD phosphatases. Mol. Cell 15, 399–407 10.1016/j.molcel.2004.06.035 15304220

[8] Almo S.C., Bonanno J.B., Sauder J.M., Emtage S., Dilorenzo T.P., Malashkevich V. et al. (2007) Structural genomics of protein phosphatases. J. Struct. Funct. Genomics 8, 121–140 10.1007/s10969-007-9036-1 18058037 PMC4163028

[9] Kashuba V.I., Li J., Wang F., Senchenko V.N., Protopopov A., Malyukova A. et al. (2004) RBSP3 (HYA22) is a tumor suppressor gene implicated in major epithelial malignancies. Proc. Natl. Acad. Sci. U.S.A. 101, 4906–4911 10.1073/pnas.0401238101 15051889 PMC387347

[10] Mitra S., Mazumder Indra D., Bhattacharya N., Singh R.K., Basu P.S., Mondal R.K. et al. (2010) RBSP3 is frequently altered in premalignant cervical lesions: clinical and prognostic significance. Genes Chromosomes Cancer 49, 155–170 19885927 10.1002/gcc.20726

[11] Kashuba V.I., Pavlova T.V., Grigorieva E.V., Kutsenko A., Yenamandra S.P., Li J. et al. (2009) High mutability of the tumor suppressor genes RASSF1 and RBSP3 (CTDSPL) in cancer. PLoS ONE 4, e5231 10.1371/journal.pone.0005231 19478941 PMC2684631

[12] Sinha S., Singh R.K., Alam N., Roy A., Roychoudhury S. and Panda C.K. (2008) Frequent alterations of hMLH1 and RBSP3/HYA22 at chromosomal 3p22.3 region in early and late-onset breast carcinoma: clinical and prognostic significance. Cancer Sci. 99, 1984–1991 19016758 10.1111/j.1349-7006.2008.00952.x PMC11158254

[13] Wang W., Liao P., Shen M., Chen T., Chen Y., Li Y. et al. (2016) SCP1 regulates c-Myc stability and functions through dephosphorylating c-Myc Ser62. Oncogene 35, 491–500 10.1038/onc.2015.106 25893300

[14] Sun T., Fu J., Shen T., Lin X., Liao L., Feng X.H. et al. (2016) The small C-terminal domain phosphatase 1 inhibits cancer cell migration and invasion by dephosphorylating Ser(P)68-Twist1 to accelerate Twist1 protein degradation. J. Biol. Chem. 291, 11518–11528 10.1074/jbc.M116.721795 26975371 PMC4882423

[15] Su Y.A., Lee M.M., Hutter C.M. and Meltzer P.S. (1997) Characterization of a highly conserved gene (OS4) amplified with CDK4 in human sarcomas. Oncogene 15, 1289–1294 10.1038/sj.onc.1201294 9315096

[16] Zhu Y., Lu Y., Zhang Q., Liu J.J., Li T.J., Yang J.R. et al. (2012) MicroRNA-26a/b and their host genes cooperate to inhibit the G1/S transition by activating the pRb protein. Nucleic Acids Res. 40, 4615–4625 10.1093/nar/gkr1278 22210897 PMC3378857

[17] Winans S., Flynn A., Malhotra S., Balagopal V. and Beemon K.L. (2017) Integration of ALV into CTDSPL and CTDSPL2 genes in B-cell lymphomas promotes cell immortalization, migration and survival. Oncotarget 8, 57302–57315 10.18632/oncotarget.19328 28915671 PMC5593642

[18] Travis W.D., Brambilla E., Nicholson A.G., Yatabe Y., Austin J.H.M., Beasley M.B. et al. (2015) The 2015 World Health Organization Classification of lung tumors: impact of genetic, clinical and radiologic advances since the 2004 classification. J. Thorac. Oncol. 10, 1243–1260 10.1097/JTO.0000000000000630 26291008

[19] Gazdar A.F., Girard L., Lockwood W.W., Lam W.L. and Minna J.D. (2010) Lung cancer cell lines as tools for biomedical discovery and research. J. Natl. Cancer Inst. 102, 1310–1321 10.1093/jnci/djq279 20679594 PMC2935474

[20] Mates L., Chuah M.K., Belay E., Jerchow B., Manoj N., Acosta-Sanchez A. et al. (2009) Molecular evolution of a novel hyperactive Sleeping Beauty transposase enables robust stable gene transfer in vertebrates. Nat. Genet. 41, 753–761 10.1038/ng.343 19412179

[21] Yegorov Y.E., Akimov S.S., Hass R., Zelenin A.V. and Prudovsky I.A. (1998) Endogenous beta-galactosidase activity in continuously nonproliferating cells. Exp. Cell Res. 243, 207–211 10.1006/excr.1998.4169 9716464

[22] Krasnov G.S., Dmitriev A.A., Melnikova N.V., Zaretsky A.R., Nasedkina T.V., Zasedatelev A.S. et al. (2016) CrossHub: a tool for multi-way analysis of The Cancer Genome Atlas (TCGA) in the context of gene expression regulation mechanisms. Nucleic Acids Res. 44, e62 10.1093/nar/gkv1478 26773058 PMC4838350

[23] Livak K.J. and Schmittgen T.D. (2001) Analysis of relative gene expression data using real-time quantitative PCR and the 2(-Delta Delta C(T)) Method. Methods 25, 402–408 10.1006/meth.2001.1262 11846609

[24] Berger A.H., Knudson A.G. and Pandolfi P.P. (2011) A continuum model for tumour suppression. Nature 476, 163–169 10.1038/nature10275 21833082 PMC3206311

[25] Croce M.V., Colussi A.G., Price M.R. and Segal-Eiras A. (1999) Identification and characterization of different subpopulations in a human lung adenocarcinoma cell line (A549). Pathol. Oncol. Res. 5, 197–204 10.1053/paor.1999.0212 10491017

[26] Narasimha A.M., Kaulich M., Shapiro G.S., Choi Y.J., Sicinski P. and Dowdy S.F. (2014) Cyclin D activates the Rb tumor suppressor by mono-phosphorylation. eLife 3, 10.7554/eLife.02872 24876129 PMC4076869

[27] Krasnov G.S., Puzanov G.A., Kudryavtseva A.V., Dmitriev A.A., Beniaminov A.D., Kondratieva T.T. et al. (2017) Differential expression of an ensemble of the key genes involved in cell-cycle regulation in lung cancer. Mol. Biol. 51, 849–856 10.1134/S0026893317050107 29116073

[28] Beniaminov A.D., Krasnov G.S., Dmitriev A.A., Puzanov G.A., Snopok B.A., Senchenko V.N. et al. (2016) Interaction of two tumor suppressors: Phosphatase CTDSPL and Rb protein. Mol. Biol. 50, 504–508 10.1134/S002689331603002X 27414789

[29] Zhang L., He X., Li F., Pan H., Huang X., Wen X. et al. (2018) The miR-181 family promotes cell cycle by targeting CTDSPL, a phosphatase-like tumor suppressor in uveal melanoma. J. Exp. Clin. Cancer Res. 37, 15 10.1186/s13046-018-0679-5 29382357 PMC5791374

[30] Lin Y.C., Lu L.T., Chen H.Y., Duan X., Lin X., Feng X.H. et al. (2014) SCP phosphatases suppress renal cell carcinoma by stabilizing PML and inhibiting mTOR/HIF signaling. Cancer Res. 74, 6935–6946 10.1158/0008-5472.CAN-14-1330 25293974

[31] Kloet D.E., Polderman P.E., Eijkelenboom A., Smits L.M., van Triest M.H., van den Berg M.C. et al. (2015) FOXO target gene CTDSP2 regulates cell cycle progression through Ras and p21(Cip1/Waf1). Biochem. J. 469, 289–298 10.1042/BJ20140831 25990325 PMC4613505

[32] Knudsen E.S. and Wang J.Y. (1996) Differential regulation of retinoblastoma protein function by specific Cdk phosphorylation sites. J. Biol. Chem. 271, 8313–8320 10.1074/jbc.271.14.8313 8626527

[33] Rubin S.M. (2013) Deciphering the retinoblastoma protein phosphorylation code. Trends Biochem. Sci. 38, 12–19 10.1016/j.tibs.2012.10.007 23218751 PMC3529988

[34] Zarkowska T. and Mittnacht S. (1997) Differential phosphorylation of the retinoblastoma protein by G1/S cyclin-dependent kinases. J. Biol. Chem. 272, 12738–12746 10.1074/jbc.272.19.12738 9139732

[35] Rubin S.M., Gall A.L., Zheng N. and Pavletich N.P. (2005) Structure of the Rb C-terminal domain bound to E2F1-DP1: a mechanism for phosphorylation-induced E2F release. Cell 123, 1093–1106 10.1016/j.cell.2005.09.044 16360038

[36] Dmitriev A.A., Kashuba V.I., Haraldson K., Senchenko V.N., Pavlova T.V., Kudryavtseva A.V. et al. (2012) Genetic and epigenetic analysis of non-small cell lung cancer with NotI-microarrays. Epigenetics 7, 502–513 10.4161/epi.19801 22491060

[37] Dambal S., Shah M., Mihelich B. and Nonn L. (2015) The microRNA-183 cluster: the family that plays together stays together. Nucleic Acids Res. 43, 7173–7188 10.1093/nar/gkv703 26170234 PMC4551935

[38] Li P., Sheng C., Huang L., Zhang H., Huang L., Cheng Z. et al. (2014) MiR-183/-96/-182 cluster is up-regulated in most breast cancers and increases cell proliferation and migration. Breast Cancer Res. 16, 473 10.1186/s13058-014-0473-z 25394902 PMC4303194

[39] Ma Y., Liang A.J., Fan Y.P., Huang Y.R., Zhao X.M., Sun Y. et al. (2016) Dysregulation and functional roles of miR-183-96-182 cluster in cancer cell proliferation, invasion and metastasis. Oncotarget 7, 42805–42825 27081087 10.18632/oncotarget.8715 PMC5173173

[40] Kundu S.T., Byers L.A., Peng D.H., Roybal J.D., Diao L., Wang J. et al. (2016) The miR-200 family and the miR-183∼96∼182 cluster target Foxf2 to inhibit invasion and metastasis in lung cancers. Oncogene 35, 173–186 10.1038/onc.2015.71 25798833 PMC4580489

[41] Dang X., Ma A., Yang L., Hu H., Zhu B., Shang D. et al. (2012) MicroRNA-26a regulates tumorigenic properties of EZH2 in human lung carcinoma cells. Cancer Genetics 205, 113–123 10.1016/j.cancergen.2012.01.002 22469510

[42] Liang N., Zhou X., Zhao M., Zhao D., Zhu Z., Li S. et al. (2015) Down-regulation of microRNA-26b modulates non-small cell lung cancer cells chemoresistance and migration through the association of PTEN. Acta Biochim. Biophys. Sin. 47, 530–538 10.1093/abbs/gmv046 26068649

[43] Liu B., Wu X., Liu B., Wang C., Liu B., Zhou Q. et al. (2012) MiR-26a enhances metastasis potential of lung cancer cells via AKT pathway by targeting PTEN. Biochim. Biophys. Acta. 1822, 1692–704 22885155 10.1016/j.bbadis.2012.07.019

[44] Dill H., Linder B., Fehr A. and Fischer U. (2012) Intronic miR-26b controls neuronal differentiation by repressing its host transcript, ctdsp2. Genes Dev. 26, 25–30 10.1101/gad.177774.111 22215807 PMC3258962

