# Supplementary material for: Tumor suppressor properties of the small C-terminal domain phosphatases in non-small cell lung cancer
Source: Biosci Rep. 2019 Dec 13;39(12):BSR20193094. doi: 10.1042/BSR20193094 (PMC6911153; doi:10.1042/BSR20193094)
Supplement: Supplementary Figures S1-S5 and Tables S1-S7 [file BSR-2019-3094_supp.pdf]

Supplementary material

Tables S1 to S7

Table S1. Clinical and pathological characteristics of samples used to extract RNA for qPCR experiments

**Characteristic:**		
**Age of patients**	39 – 81 (mean 59)	
**TNM/Stage**	Histological type of NSCLC (no. of patients)	
	SCC	ADC
**$T_1N_0M_0$, $T_2N_0M_0$/Stage (IA+ IB)**	10	5
**$T_3N_0M_0$, $T_2N_1M_0$/Stage IIB**	10	1
**$T_1N_2M_0$, $T_2N_2M_0$, $T_3N_1M_0$, $T_3N_2M_0$/Stage IIIA**	13	5
**$T_4N_0M_0$/Stage IIIB**	–	2
**N_0 Stage (no metastases)**	13	7
**N_1 Stage +N_2 Stage (with metastases)**	20	6
**Total:**	**33**	**13**

Note: SCC – Squamous Cell Carcinoma*;* ADC – adenocarcinoma.

Table S2. Primers and TaqMan probes for qPCR.

**Gene**	**Primers (F, R) and probe (Z) sequences 5'→3'**		**Amplicon length**
CTDSPL (cDNA)	F	CCACCATTCTCCTCCACCAGT	154
	R	CCACCATTCTCCTCCACCAGT	
	Z	FAM-CCACATTGTAATCACGGAAGCAGCAGA-BHQ1	
CTDSPL (DNA)	F	CAGAGTGCGTGTGCCGACT	126
	R	ACAACTTCTCTGCGGGCGT	
	Z	FAM-CTGGCGGAGAGACTGGGAGCGA-BHQ1	
ACTB (DNA)	F	GTGCTCAGGGCTTCTTGTCCTTT	160
	R	TTTCTCCATGTCGTCCCAGTTGGT	
	Z	FAM-AAGGATTCCTATGTGGGCGACGAGGCCCA-BHQ1	
RPN1 (cDNA)	F	CACCCTCAACAGTGGCAAGAAG	125
	R	TGCATTTCGCTCACTCTGTCG	
	Z	FAM-CCCTCTGTCTTCAGCCTGGACTGC-BHQ1	

Note: F – forward primer; R – reverse primer; Z – probe.

Table S3. **Differential expression of *CTDSP1/2/L/L2* and *RB1* in non-small cell lung cancer according to TCGA data.**

	Pooled samples							Paired samples					
Gene Name	Log2FC	FDR	P-value	Rel. StDev(T), %	Rel. StDev(N), %	Tumor CPM	Norm CPM	Log2FC (mean)	FDR	P-value	Tumor CPM	Norm CPM	
CTDSPL	-1,2	5,03E-52	1,01E-52	45,0	27,9	43,5	101,2	-1,0	1,39E-11	2,78E-12	50,9	101,2	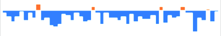
CTDSP1	-0,6	1,25E-18	5,01E-19	33,0	16,7	118,0	176,1	-0,6	2,38E-09	9,51E-10	122,1	176,1	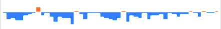
CTDSPL2	0,6	1,38E-07	8,27E-08	45,2	20,8	44,6	29,3	0,6	1,61E-07	9,64E-08	46,8	29,3	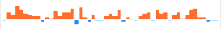
CTDSP2	-0,4	3,28E-05	2,62E-05	49,3	22,0	272,4	368,0	-0,3	0,000368	0,000295	306,5	368,0	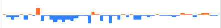
RB1	0,0	0,85922	0,85922	48,0	21,4	61,4	60,8	-0,3	0,951593	0,951593	62,1	60,8	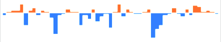

(**A**) Relative expression level of *CTDSP1/2/L/L2* and *RB1* genes in lung squamous cell carcinomas (TCGA LUSC dataset). Log2FC – binary logarithm of expression level fold change (tumor versus normal); FDR – false discovery rate; Rel. StDev. - relative standard deviation (also named CV, coefficient of variation) of the expression levels; CMP – read counts per million.

	Pooled samples							Paired samples					
Gene Name	Log2FC	FDR	P-value	Rel. StDev(T), %	Rel. StDev(N), %	Tumor CPM	Norm CPM	Log2FC (mean)	FDR	P-value	Tumor CPM	Norm CPM	
CTDSPL	-1,1	4,81E-40	9,62E-41	52,2	21,6	54,0	118,9	-1,2	1,55E-19	3,11E-20	50,8	118,1	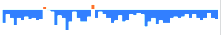
CTDSP1	-0,3	1,33E-08	5,3E-09	29,1	15,2	148,4	186,2	-0,5	2,16E-10	8,66E-11	136,9	186,1	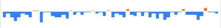
RB1	-0,4	1,71E-08	1,02E-08	41,2	19,7	46,8	63,5	-0,4	1,3E-05	7,82E-06	52,0	63,3	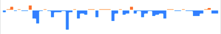
CTDSP2	-0,4	0,000183	0,000147	51,5	17,0	284,1	383,0	-0,3	0,126101	0,100881	291,9	382,2	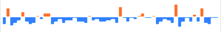
CTDSPL2	-0,1	0,779243	0,779243	38,6	19,0	29,4	30,6	0,0	0,424956	0,424956	31,9	30,7	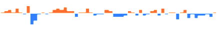

(**B**) Relative expression level of *CTDSP1/2/L/L2* and *RB1* genes in lung adenocarcinoma (TCGA LUAD dataset). Log2FC – binary logarithm of expression level fold change (tumor versus normal); FDR – false discovery rate; Rel. StDev. - relative standard deviation (also named CV, coefficient of variation) of the expression levels; CMP – read counts per million.

Table S4. **Differential expression of mir-26a/b in non-small cell lung cancer according to TCGA data.**

	Pooled samples							Paired samples					
miRNA	Log2FC	FDR	P-value	Rel. StDev(T) %	Rel. StDev(N) %	Tumor CPM	Norm CPM	Log2FC (mean)	FDR	P-value	Tumor CPM	Norm CPM	Log2FC profile
hsa-mir-26a-2	-1,3	5,96E-30	1,2E-31	53,9	55,5	2762,9	6624,1	-1,1	4,89E-10	1,03E-11	3740,0	7612,0	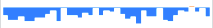
hsa-mir-26b	-0,3	0,822456	0,172983	51,4	41,9	626,8	761,3	-0,8	3,71E-08	1,06E-09	497,9	849,5	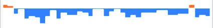
hsa-mir-26a-1						1,0	0,6				0,8	0,6	discarded. too few reads

(**A**) Relative expression level of mir-26a/b in lung squamous cell carcinomas (TCGA LUSC dataset). Log2FC – binary logarithm of expression level fold change (tumor versus normal); FDR – false discovery rate; Rel. StDev. - relative standard deviation (also named CV, coefficient of variation) of the expression levels; CMP – read counts per million.

	Pooled samples							Paired samples					
miRNA	Log2FC	FDR	P-value	Rel. StDev(T) %	Rel. StDev(N) %	Tumor CPM	Norm CPM	Log2FC (mean)	FDR	P-value	Tumor CPM	Norm CPM	Log2FC profile
hsa-mir-26a-2	-1,4	4,11E-66	3,54E-68	38,9	31,9	1908,0	5136,9	-1,6	1,35E-12	1,29E-14	1757,8	5136,9	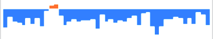
hsa-mir-26b	-0,5	5,93E-05	6,46E-06	47,3	31,1	610,9	851,4	-0,8	0,000164	1,92E-05	506,8	851,4	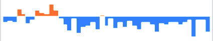
hsa-mir-26a-1						1,4	1,3				1,2	1,3	discarded. too few reads

(**B**) Relative expression level of mir-26a/b in lung adenocarcinoma (TCGA LUAD dataset). Log2FC – binary logarithm of expression level fold change (tumor versus normal); FDR – false discovery rate; Rel. StDev. - relative standard deviation (also named CV, coefficient of variation) of the expression levels; CMP – read counts per million.

Table S5. Summary of qPCR data for *CTDSPL*, *CTDSP1*, *CTDSP2* and *RB1* genes in non-small cell lung cancer (NSCLC: ADC and SCC).

**Quantitative characteristics**	**ADC**	**SCC**	**ADC**	**SCC**
	CTDSPL		***RB1***	
Average mRNA level fold change	6.7↓[a]	6.8↓[a]	4.6↓[a]	3.0↓[a]
FD, %	77	72	69	59
LD_{av}, n-fold	8.3↓	8.9↓	6↓	4.3↓
FI, %	no	3	7	3
LI_{av}, n-fold	no	2	2.5	2.5
Average mRNA level for groups without and with metastasis	2.6↓ (no met)	3.9↓ (no met)	1.7↓ (no met)	2.6↓ (no met)
	11.6↓ (met)	8.7↓ (met)	7.9↓ (met)	3.3↓ (met)
	$P < 0.01$	$P = 0.62$	$P < 0.01$	$P = 0.66$
	CTDSP1		***CTDSP2***	
Average mRNA level for all samples, n-fold	3.6↓[a]	3.5↓[a]	5.2↓[a]	4.0↓[a]
FD, %	64	68	90	70
LD_{av}, n-fold	4.6↓	4.5↓	5.7↓	5.2↓
FI, %	no	no	no	no
LI_{av}, n-fold	no	no	no	no
Average mRNA level for groups without and with metastasis	2.0↓ (no met)	2.4↓ (no met)	4.6↓ (no met)	2.5↓ (no met)
	5.3↓ (met)	4.2↓ (met)	5.8↓ (met)	5.0↓ (met)
	$P < 0.05$	$P = 0.27$	$P < 0.06$	$P = 0.59$

Note: LD_{av}, average mRNA level decrease; LI_{av}, average mRNA level increase; FD, frequency of decrease; FI, frequency of increase; ↑, increase; ↓, decrease; met, metastases; ADC (N=13) and SCC (N=33); [a]$P < 0.02$ for each value.

Table S6. Top scored common regulatory microRNAs for SCP subfamily genes (lung SCC). Orange background highlights the members of oncogenic mir-96/182/183 family, which are overexpressed in NSCLC.

	CTDSPL2	*RB1*	*CTDSP2*	*CTDSP1*	*CTDSPL*				
LogFC [pooled]	-0,1	-0,4	-0,4	-0,3	-1,1				
FDR	0,779243	1,71E-08	0,000183	1,33E-08	4,81E-40				
avg. CPM	30,0	55,2	333,6	167,3	86,4				
LogFCs						LogFC	FDR	avg. CPM	LogFCs
hsa-mir-183	0,11	-0,20	-0,14	-0,24	-0,27	2,2	2,5E-07	6288,7	
hsa-mir-20a	-0,20	-0,31	-0,26	0,03	-0,26	0,9	8,7E-05	173,3	
hsa-mir-93	-0,12	-0,43	-0,27	-0,14	-0,35	1,2	6,33E-07	2744,3	
hsa-mir-182	0,05	-0,19	-0,14	-0,10	-0,16	1,9	4,41E-10	13736,6	
hsa-mir-9-2	0,01	-0,24	-0,20	-0,31	-0,42	4,7	0,311093	380,6	
hsa-mir-9-1	0,01	-0,24	-0,20	-0,31	-0,41	4,7	0,311093	380,7	
hsa-mir-96	-0,18	-0,38	-0,28	-0,10	-0,34	2,6	5,43E-09	13,9	
hsa-mir-17	0,04	-0,13	-0,17	0,03	-0,15	0,4	0,00715	597,3	
hsa-mir-29b-1	-0,46	-0,19	-0,22	0,14	-0,12	1,1	1,7E-06	317,9	
hsa-mir-30b	-0,36	-0,25	-0,03	0,22	0,20	-0,6	0,012228	666,3	
hsa-mir-34a	-0,48	-0,32	-0,08	0,15	0,01	0,7	2,26E-05	173,8	
hsa-mir-424	0,00	-0,09	-0,10	-0,19	-0,30	1,2	0,003977	54,4	
hsa-mir-142	-0,19	-0,14	-0,19	-0,23	-0,34	1,7	6,98E-08	1446,6	
hsa-mir-106a	-0,11	-0,23	-0,26	-0,09	-0,30	0,7	0,058244	8,7	
hsa-mir-130b	-0,01	-0,28	-0,16	-0,32	-0,42	1,6	0,000611	20,3	
hsa-mir-590	-0,01	-0,26	-0,33	-0,23	-0,51	1,0	3,16E-06	12,0	
hsa-mir-128-1	0,34	0,02	-0,11	-0,27	-0,31	0,4	0,01125	70,2	
hsa-mir-26b	-0,15	-0,05	-0,11	0,31	0,02	-0,3	0,822456	694,0	
hsa-mir-429	-0,12	-0,27	-0,15	0,09	-0,01	1,1	3,7E-05	84,7	
hsa-mir-106b	0,23	-0,04	-0,05	-0,16	-0,22	0,1	1	581,5	
hsa-mir-21	-0,19	-0,14	-0,07	-0,01	-0,20	1,8	1,83E-18	229334,6	
hsa-mir-7-1	0,16	-0,04	-0,18	-0,06	-0,21	1,4	5,23E-07	13,7	
hsa-mir-199a-1	-0,22	-0,12	-0,01	-0,07	-0,08	0,8	8,13E-07	788,9	
hsa-mir-128-2	0,36	0,06	-0,12	-0,26	-0,30	0,4	0,013333	54,9	
hsa-mir-186	-0,03	-0,22	-0,22	0,02	-0,17	0,3	0,002261	233,4	
hsa-mir-22	-0,17	0,08	-0,08	0,09	-0,04	0,2	0,013516	69864,3	
hsa-mir-135b	-0,22	-0,11	-0,08	0,28	0,12	2,5	1,22E-06	33,4	
hsa-mir-199a-2	-0,19	-0,10	-0,01	-0,10	-0,10	0,7	1,82E-06	1305,3	
hsa-mir-193b	-0,13	-0,18	-0,12	-0,01	-0,32	1,6	0,00665	41,1	
hsa-mir-19a	-0,28	-0,37	-0,27	0,02	-0,31	1,1	0,001552	14,7	
hsa-mir-141	-0,05	-0,09	-0,18	0,19	-0,13	1,5	1,65E-10	1029,6	
hsa-mir-335	0,06	-0,04	-0,11	-0,16	-0,14	-0,1	1	119,9	
hsa-mir-33a	-0,09	-0,20	-0,25	-0,15	-0,25	1,3	2,13E-05	13,4	
hsa-mir-155	-0,11	0,02	-0,01	-0,19	-0,10	0,2	0,626623	383,4	
hsa-mir-199b	-0,16	-0,08	-0,01	-0,12	-0,11	0,7	1,15E-05	1652,4	
hsa-mir-210	-0,11	-0,27	-0,21	-0,12	-0,42	5,1	1,23E-08	479,9	
hsa-mir-30d	-0,10	-0,04	0,11	0,22	0,40	-1,1	8,11E-11	18255,4	
hsa-mir-194-2	-0,06	-0,01	-0,16	0,15	0,03	1,2	0,546912	134,1	
hsa-mir-29b-2	-0,47	-0,21	-0,21	0,16	-0,10	1,1	2,01E-06	343,1	
hsa-mir-20b	-0,10	-0,28	-0,21	-0,15	-0,37	0,7	0,278212	10,8	

Comments: Top-40 potential regulating microRNA are listed. The values in each cell represent coefficient of Spearman correlation between the gene and the microRNA expression level. The bars inside cells (regardless of color) indicate prediction score of microRNA binding site according to several databases (from left to right): miRTarBase (strong experimental evidence), TargetScan (conservative binding sites, CS), PicTar (CS), DIANA microT, mirSVR (CS), TargetScan (non-CS), PicTar (non-CS), miRTarBase (weak experimental evidence), mirSVR (non-CS). Cell borders indicate miRNA-gene pairs with predicted binding site (dashed border – only non-CS sites; double border – several reliable algorithms). On the right side of the figure, expression profiles of microRNAs in paired tumor-normal samples are presented in logarithmic scale (from -2 to +2, e.g. from 4-fold decrease to 4-fold increase). MicroRNAs are sorted by overall prediction score (decreasing).

Table S7. Top scored common regulatory microRNA for SCP subfamily genes (lung ADC). Orange background highlights the members of oncogenic mir-96/182/183 family, which are overexpressed in NSCLC.

	CTDSPL2	*RB1*	*CTDSP2*	*CTDSP1*	*CTDSPL*				
LogFC	0,6	0,0	-0,4	-0,6	-1,2				
FDR	1,38E-07	0,85922	3,28E-05	1,25E-18	5,03E-52				
avg. CPM	36,9	61,1	320,2	147,1	72,3				
LogFCs						LogFC	FDR	avg. CPM	LogFCs
hsa-mir-183	0,30	-0,06	-0,17	-0,27	-0,27	2,5	3,77E-09	7902,0	
hsa-mir-182	0,20	-0,08	-0,18	-0,21	-0,22	1,8	7,43E-07	15679,5	
hsa-mir-96	0,20	-0,12	-0,29	-0,22	-0,26	2,6	8,94E-08	18,4	
hsa-mir-30b	-0,34	-0,15	-0,07	0,29	0,24	-2,0	1,28E-54	864,0	
hsa-let-7a-1	-0,16	0,04	0,19	0,17	0,28	-1,3	2,75E-47	10392,0	
hsa-let-7a-3	-0,16	0,04	0,19	0,17	0,28	-1,3	3,89E-47	10439,5	
hsa-mir-9-2	0,27	-0,11	-0,21	-0,27	-0,31	5,3	0,001594	1239,4	
hsa-mir-9-1	0,27	-0,11	-0,21	-0,27	-0,31	5,3	0,001594	1240,6	
hsa-mir-30a	-0,32	0,00	0,19	0,21	0,28	-3,5	1,1E-107	82918,9	
hsa-mir-26b	-0,32	-0,13	-0,06	0,49	0,10	-0,5	5,93E-05	731,1	
hsa-mir-26a-2	-0,35	-0,08	0,26	0,25	0,27	-1,4	4,11E-66	3522,5	
hsa-mir-22	-0,33	0,12	0,17	0,11	0,31	-0,9	2,06E-35	135077,1	
hsa-mir-29a	-0,44	-0,07	0,13	0,25	0,23	-1,5	1,06E-45	11668,1	
hsa-mir-149	0,28	0,04	-0,17	0,08	-0,15	2,8	4,76E-08	116,4	
hsa-mir-30d	-0,23	-0,09	0,14	0,19	0,27	-2,7	2,53E-93	21762,1	
hsa-let-7g	-0,19	-0,07	0,02	0,10	0,14	-0,4	4,49E-06	554,3	
hsa-mir-7-1	0,31	-0,10	-0,19	-0,24	-0,20	1,0	6,16E-06	22,1	
hsa-mir-486	-0,17	-0,01	0,13	0,19	0,28	-3,9	9,56E-58	852,1	
hsa-mir-132	-0,19	-0,02	0,17	-0,01	0,13	-0,3	0,039192	123,6	
hsa-mir-15b	0,08	-0,07	-0,27	-0,15	-0,09	0,1	1	288,8	
hsa-let-7e	-0,15	-0,14	0,28	0,15	0,14	-0,7	1,99E-06	1169,6	
hsa-mir-34a	-0,32	-0,12	-0,05	0,18	0,22	-0,9	1,27E-21	239,4	
hsa-mir-93	0,28	-0,02	-0,30	-0,26	-0,23	0,8	5,08E-05	7120,1	
hsa-mir-192	0,09	-0,01	-0,08	-0,19	-0,07	0,3	1	369,5	
hsa-mir-143	-0,22	0,10	0,27	0,14	0,29	-2,0	2,51E-47	124933,5	
hsa-mir-139	-0,36	-0,03	0,10	0,16	0,27	-2,9	1,65E-68	130,6	
hsa-mir-374b	-0,24	-0,23	0,02	0,18	0,14	-1,0	3,86E-27	87,3	
hsa-mir-29b-1	-0,39	-0,15	-0,12	0,12	-0,03	-0,4	0,22746	375,7	
hsa-mir-424	-0,04	-0,02	-0,09	-0,02	-0,16	1,0	2,24E-05	52,7	
hsa-mir-10a	-0,22	-0,01	0,26	0,20	0,17	-1,2	1,09E-16	36539,0	
hsa-let-7b	-0,09	0,12	0,30	0,23	0,37	-1,3	2,7E-30	24275,7	
hsa-mir-224	0,15	0,06	-0,22	-0,28	-0,17	2,5	0,000139	93,0	
hsa-mir-155	-0,22	-0,02	-0,06	-0,05	-0,11	0,1	1	469,1	
hsa-mir-101-2	-0,31	-0,05	0,03	0,11	0,23	-2,1	2,15E-58	100,2	
hsa-mir-338	-0,31	-0,03	0,00	0,19	0,16	-2,9	7,8E-44	1863,0	
hsa-mir-130b	0,40	-0,04	-0,12	-0,22	-0,27	2,2	3,57E-12	30,8	
hsa-mir-20a	0,10	-0,01	-0,23	-0,15	-0,08	0,4	0,105018	448,4	
hsa-mir-141	0,18	0,02	-0,22	-0,10	-0,20	1,4	6,64E-09	1420,0	
hsa-mir-128-1	0,39	-0,02	-0,10	-0,29	-0,11	0,5	0,015337	113,8	
hsa-mir-193a	-0,21	0,09	0,24	0,06	0,20	-0,5	0,005131	279,3	

Comments: Top-40 potential regulating microRNA are presented. The values in each cell represent coefficient of Spearman correlation between gene and microRNA expression level. The bars inside cells (regardless of color) indicate prediction score of microRNA binding site according to several databases (from left to right): miRTarBase (strong experimental evidence), TargetScan (conservative binding sites, CS), PicTar (CS), DIANA microT, mirSVR (CS), TargetScan (non-CS), PicTar (non-CS), miRTarBase (weak experimental evidence), mirSVR (non-CS). Cell borders indicate miRNA-gene pairs with predicted binding site (dashed border – only non-CS sites; double border – several reliable algorithms). On the right side of the figure, expression profiles of microRNAs in paired tumor-

normal samples are presented in logarithmic scale (from -2 to +2, e.g. from 4-fold decrease to 4-fold increase). MicroRNAs are sorted by overall prediction score (decreasing).

Figures S1 to S5

```
sp|Q9GZU7|CTDSP1    MDSS---------AVITQI-----SK-E-----------------------------------
sp|O14595|CTDSP2    MEHG---------SIITQA-----RR-------------------------------------
sp|O15194|CTDSPL    MDGP---------AIITQV-----TNPK-----------------------------------
sp|Q05D32|CTDSPL2   MRLRTRKASQQSNQIQTQRTARAKRKYSEVDDSLPSGGEKPSKNETGLLSSIKKFIKGSTPKE
cons                *             :  **       .

sp|Q9GZU7|CTDSP1    --------------------------------------------EARGPLRGKGDQKSAASQ-
sp|O14595|CTDSP2    --------------------------------------------EDALVLTKQGLVSKSSPK-
sp|O15194|CTDSPL    --------------------------------------------EDEGRLPGAGEKASQCNVS
sp|Q05D32|CTDSPL2   ERENPSKRSRIERDIDNNLITSTPRAGEKPNKQISRVRRKSQVNGEAGSYEMTNQHVKQNGKL
cons                                                                     .   .

sp|Q9GZU7|CTDSP1    --K------PRS---RGILHSLFCCVCRDDGEALPAHSGA--PLLVEE---------------
sp|O14595|CTDSP2    --K------PRG---RNIFKALFCCFRAQHVGQSSSSTEL--AAYKEE---------------
sp|O15194|CTDSPL    LKK------QRS---RSILSSFFCCFRDYNVEAPPPSSPSVLPPLVEE---------------
sp|Q05D32|CTDSPL2   EDNPSSGSPPRTTLLGTIFSPVFNFFSPANKNGTSGSDSPGQAVEAEEIVKQLDMEQVDEITT
cons                  :       *      *: ..*  .   .    .       .   **

sp|Q9GZU7|CTDSP1    ----------------------NGAIPK---------Q---TP----VQ--------------
sp|O14595|CTDSP2    ----------------------ANTIAKSDLLQCLQYQFYQIP----GT--------------
sp|O15194|CTDSPL    ----------------------NGGLQKGDQRQVIPIP--SPP----AK--------------
sp|Q05D32|CTDSPL2   STTTSTNGAAYSNQAVQVRPSLNNGLEEAEETVNRDIPPLTAPVTPDSGYSSAHAEATYEEDW
cons                                       . : :              *

sp|Q9GZU7|CTDSP1    ------YLL---PEAK-------------AQDSDKICVVIDLDETLVHSSFKPVNNADFIIPV
sp|O14595|CTDSP2    ------CLL---PEVT-------------EEDQGRICVVIDLDETLVHSSFKPINNADFIVPI
sp|O15194|CTDSPL    ------YLL---PEVT-------------VLDYGKKCVVIDLDETLVHSSFKPISNADFIVPV
sp|Q05D32|CTDSPL2   EVFDPYYFIKHVPPLTEEQLNRKPALPLKTRSTPEFSLVLDLDETLVHCSLNELEDAALTFPV
cons                       ::   *  .               .  . .:*:********.*:: :.:* : .*:

sp|Q9GZU7|CTDSP1    EIDGVVHQVYVLKRPHVDEFLQRMGELFECVLFTASLAKYADPVADLLDK-WGAFRARLFRES
sp|O14595|CTDSP2    EIEGTTHQVYVLKRPYVDEFLRRMGELFECVLFTASLAKYADPVTDLLDR-CGVFRARLFRES
sp|O15194|CTDSPL    EIDGTIHQVYVLKRPHVDEFLQRMGQLFECVLFTASLAKYADPVADLLDR-WGVFRARLFRES
sp|Q05D32|CTDSPL2   LFQDVIYQVYVRLRPFFREFLERMSQMYEIILFTASKKVYADKLLNILDPKKQLVRHRLFREH
cons                 ::.. :****  **.. ***.**.:::* :*****   *** : ::**     .* *****

sp|Q9GZU7|CTDSP1    CVFHRGNYVKDLSRLGRDLRRVLILDNSPASYVFHPDNAVPVASWFDNMSDTELHDLLPFFEQ
sp|O14595|CTDSP2    CVFHQGCYVKDLSRLGRDLRKTLILDNSPASYIFHPENAVPVQSWFDDMADTELLNLIPIFEE
sp|O15194|CTDSPL    CVFHRGNYVKDLSRLGRELSKVIIVDNSPASYIFHPENAVPVQSWFDDMTDTELLDLIPFFEG
sp|Q05D32|CTDSPL2   CVCVQGNYIKDLNILGRDLSKTIIIDNSPQAFAYQLSNGIPIESWFMDKNDNELLKLIPFLEK
cons                **  :* *:***. ***:* :.:*:**** :: :: .*.:*: *** :  *.** .*:*::*

sp|Q9GZU7|CTDSP1    LSRVD-DVYSVL------RQPRPGS
sp|O14595|CTDSP2    LSGAE-DVYTSL------GQLRA-P
sp|O15194|CTDSPL    LSRED-DVYSML------HRLCN-R
sp|Q05D32|CTDSPL2   LVELNEDVRPHIRDRFRLHDLLPPD
cons                *   : ** . :

Alignment score: BAD AVG GOOD
```

Figure S1. Multiple sequence alignment of the active site regions of CTDSP1/2/L/L2 proteins (performed by T-COFFEE, version 11). Color indicates alignments score (from deep purple to light red). The following UniProt sequences have been used: Q9GZU7 (CTDSP1), O14595 (CTDSP2), O15194 (CTDSPL), Q05D32 (CTDSPL2).

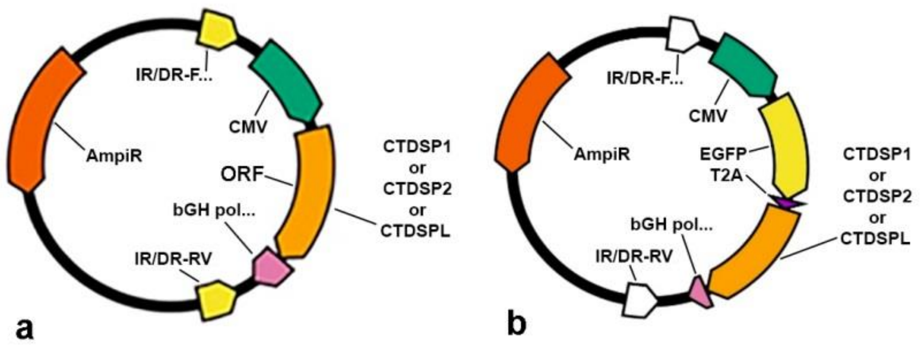

Figure S2. Plasmid design. (**a**) Plasmids pT2/HB-CMV-CTDSP1, -CTDSP2, or -CTDSPL (5168bp, 5201bp, or 5261 bp). IR/DR-FW sequence: 117-347; CMV promoter: 464-1049; open reading frame: 1006-1807, 1006-1840, or 1006-1900 (CTDSP1, CTDSP2, or CTDSPL, accordingly); bGHpA polyadenylation signal: 1834-2037 (+33 or +93 for CTDSP2 or CTDSPL); IR/DR-RV sequence: 2115-2326 (+33, +93 for CTDSP2, CTDSPL); Ampicillin Resistance Gene: 3629-4489 (+33, +93 for CTDSP2, CTDSPL). (**b**) Scheme of genetic constructs pT2 / HB-CMV-EGFP-2A-CTDSP1 (5951 bp), pT2 / HB-CMV-EGFP-2A-CTDSP2 (5984 bp), and pT2 / HB-CMV-EGFP-2A-CTDSPL (6044 bp).

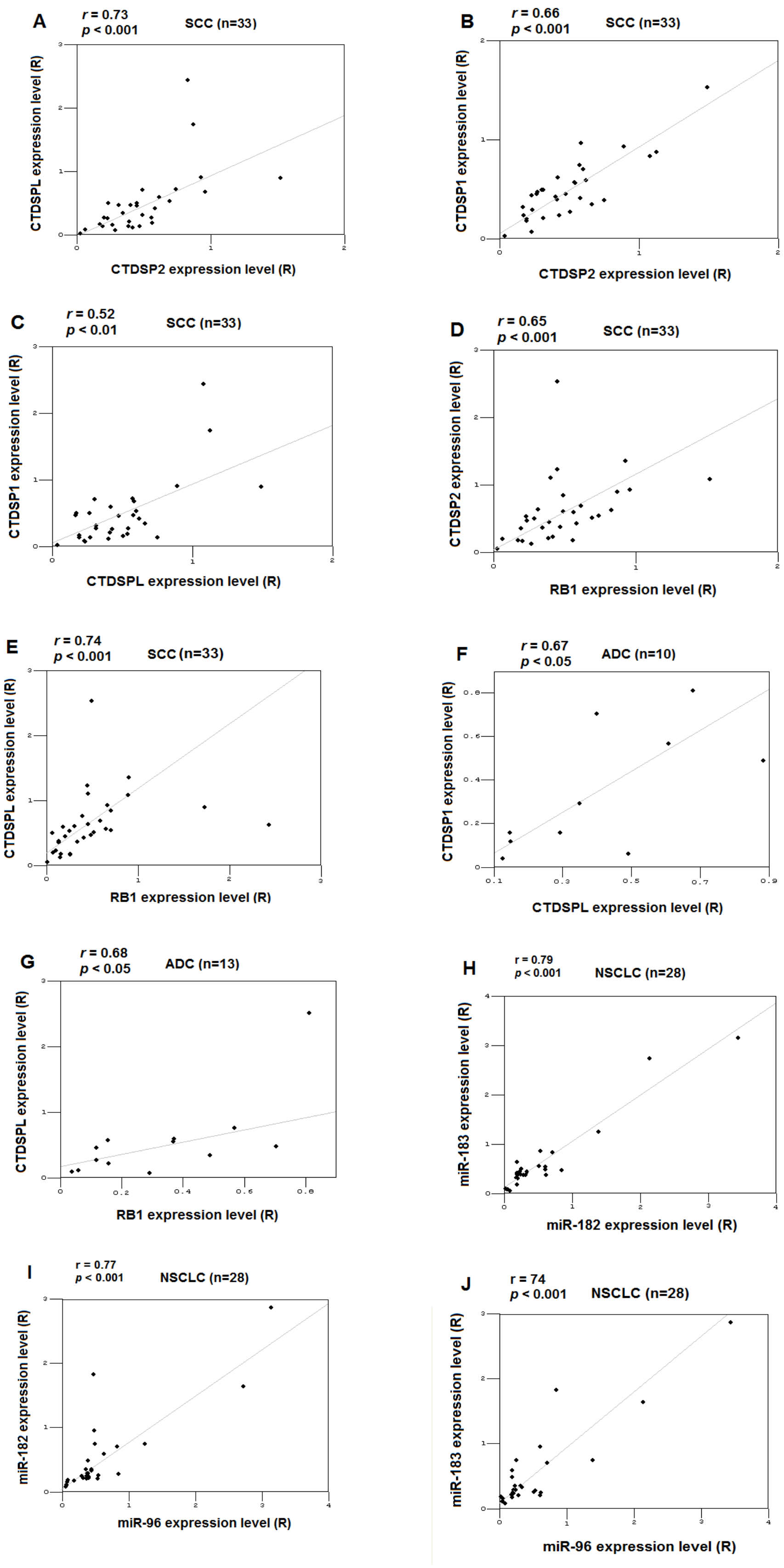

Figure S3. The values of correlation coefficients (r_S) between the mRNA levels of CTDSP1/2/L and RB1 genes in primary NSCLC samples: SCC (**A-E**) and ADC (**F, G**) and correlation between miR-183, miR-96 and miR-182 expression in NSCLC samples (**H-J**).

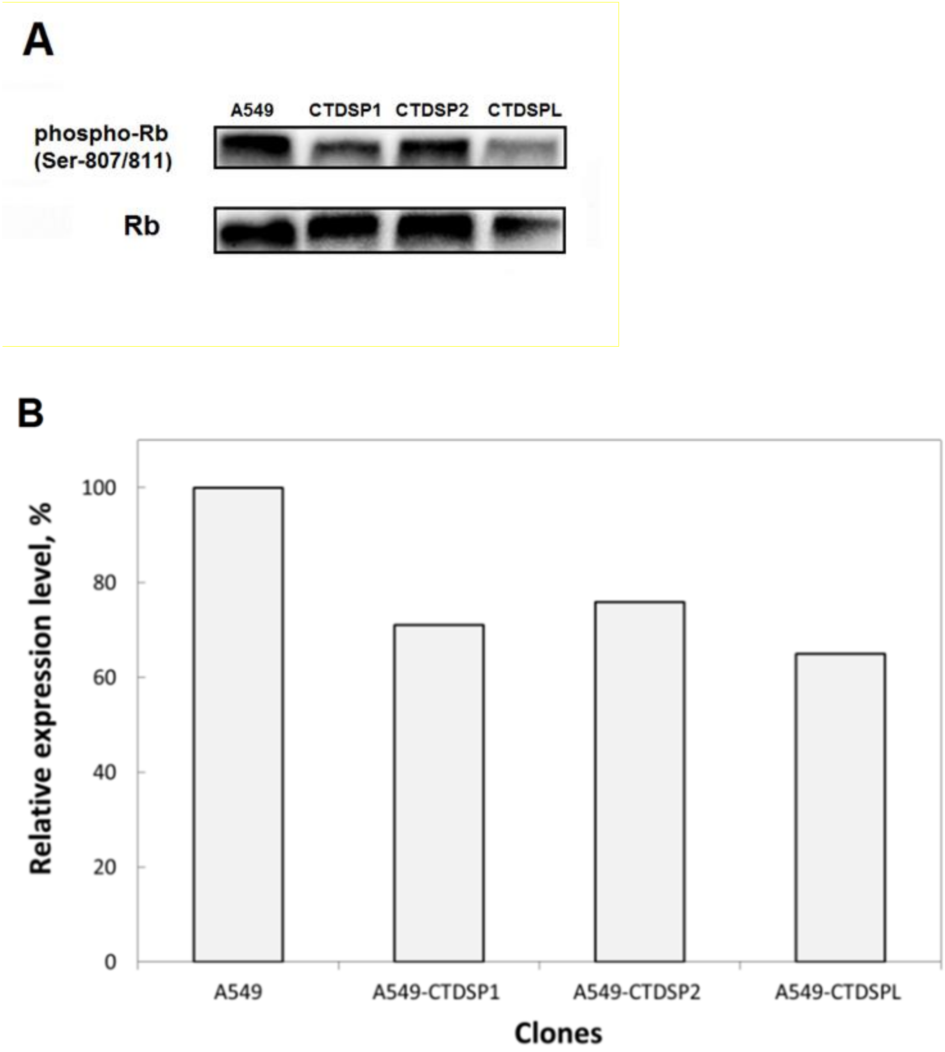

Figure S4. Alterations in the phosphorylation level of Rb at Ser-807/811 in A549 cells transfected with *CTDSP1*, *CTDSP2*, or *CTDSPL*. (**A**) Western blot showing phospho-Rb and total Rb content in selected clones of A549 cells transfected with pT2/HB-CMV-CTDSP1, 2, or L. (**B**) Quantification of Western blot experiment. The plot demonstrates the relative phosphorylation level of Rb at Ser-807/811 in the resulting clones compared with the non-transfected A549 cells. For each clone, the density of the phospho-Rb band was divided by the density of the total Rb band and normalized to the non-transfected A549 value. The measurement of target proteins was carried out twice. Band intensity was determined using ImageJ software.

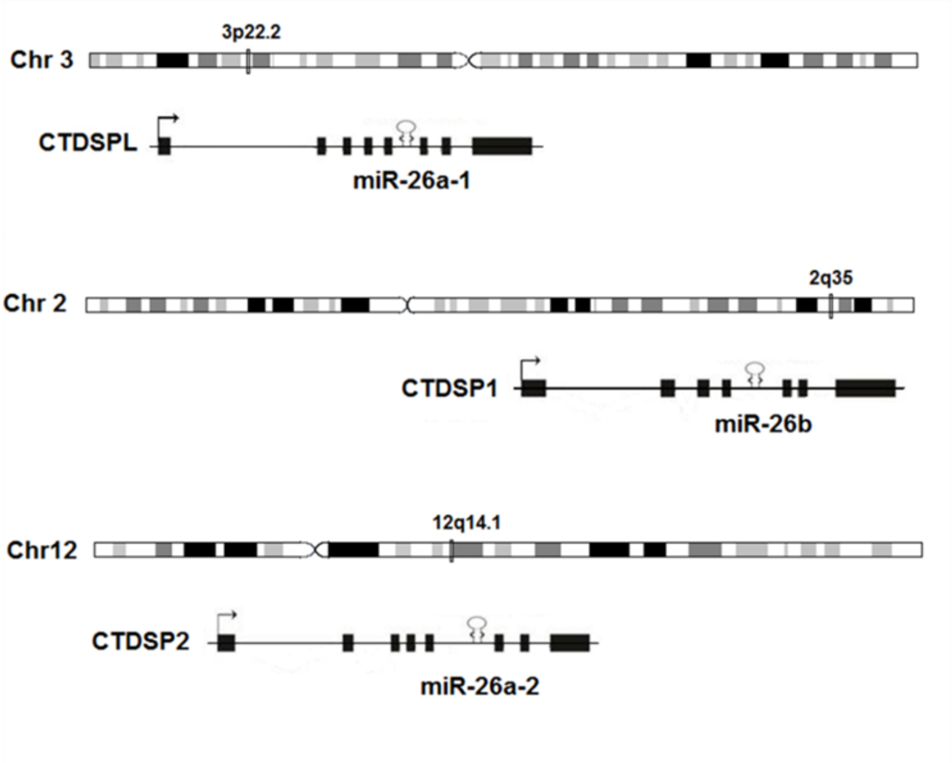

Figure S5. Genomic localization of SCP phosphatases genes and miR26a/b.
